# Establishment of a lncRNA-Based Prognostic Gene Signature Associated With Altered Immune Responses in HCC

**DOI:** 10.3389/fimmu.2022.880288

**Published:** 2022-04-28

**Authors:** Xiawei Li, Zhiqian Zhang, Mingcheng Liu, Xing Fu, Jun A, Guoan Chen, Shian Wu, Jin-Tang Dong

**Affiliations:** ^1^Department of Genetics and Cell Biology, College of Life Sciences, Nankai University, Tianjin, China; ^2^Laboratory Department of Human Cell Biology and Genetics, School of Medicine, Southern University of Science and Technology, Shenzhen, China

**Keywords:** hepatocellular carcinoma (HCC), T cell exclusion, lncRNA, prognosis, *LINC01134*, *AC116025.2*

## Abstract

Hepatocellular carcinoma (HCC) is a common malignancy with higher mortality, and means are urgently needed to improve the prognosis. T cell exclusion (TCE) plays a pivotal role in immune evasion, and lncRNAs represent a large group of tumor development and progression modulators. Using the TCGA HCC dataset (n=374), we identified 2752 differentially expressed and 702 TCE-associated lncRNAs, of which 336 were in both groups. As identified using the univariate Cox regression analysis, those associated with overall survival (OS) were subjected to the LASSO-COX regression analysis to develop a prognosis signature. The model, which consisted of 11 lncRNAs and was named 11LNCPS for 11-lncRNA prognosis signature, was validated and performed better than two previous models. In addition to OS and TCE, higher 11LNCPS scores had a significant correlation with reduced infiltrations of CD8+ T cells and dendritic cells (DCs) and decreased infiltrations of Th1, Th2, and pro B cells. As expected, these infiltration alterations were significantly associated with worse OS in HCC. Analysis of published data indicates that HCCs with higher 11LNCPS scores were transcriptomically similar to those that responded better to PDL1 inhibitor. Of the 11LNCPS lncRNAs, *LINC01134* and *AC116025.2* seem more crucial, as their upregulations affected more immune cell types’ infiltrations and were significantly associated with TCE, worse OS, and compromised immune responses in HCC. LncRNAs in the 11LNCPS impacted many cancer-associated biological processes and signaling pathways, particularly those involved in immune function and metabolism. The 11LNCPS should be useful for predicting prognosis and immune responses in HCC.

## Introduction

Hepatocellular carcinoma (HCC) is one of the most common human malignancies and the third leading cause of cancer-associated deaths worldwide (1, 2). Several therapies such as surgical resection, liver transplantation, radiotherapy, and chemotherapy are available for HCC treatment. However, the survival of patients with advanced or metastatic HCC is quite limited, and the lack of timely diagnosis, prognosis evaluation, and effective treatments are some of the reasons (3, 4). It is thus imperative to develop prognostic models that can help decision making in HCC treatment.

Accumulating evidence indicates that immunotherapy is a promising strategy for cancer treatment, which largely relies on the successful application of immune-checkpoint inhibitors (ICIs) at present (1, 5–7). The combination of ICIs and conventional therapies are also under development as additional therapeutic strategies for HCC treatment (8). For instance, combined administration of the PDL1 inhibitor atezolizumab and the VEGF inhibitor bevacizumab has become a first-line therapeutic strategy for advanced HCC (9). Although immunotherapy has shown remarkable outcomes, only one-third of patients benefit from it (10). One of the main factors affecting the effectiveness of immunotherapy is tumor immune evasion (11, 12). Cancer cells evade the immune system to avoid antitumor immunity and enhance tumor malignancy (1, 13, 14), and T cell exclusion (TCE) is one of the primary mechanisms for tumor immune escape (15). Some immunosuppressive factors exclude T cells, especially cytotoxic CD8+ T cells, from infiltration tumors, making a tumor “cold”. Hence, it is crucial to construct accurate prognostic models for TCE in HCC, which could help predict patient response to immunotherapy.

Long noncoding RNAs (lncRNAs) are a common type of noncoding RNAs with more than 200 nucleotides in length and play essential roles in cancer development and progression (1, 16–18). For example, lncRNAs regulate cancer progression by changing the transcriptome and proteome of cancer cells and influencing the infiltration of immune cells to alter the immune microenvironment (19–21). LncRNAs could thus act as immune regulators in tumor immune evasion. Therefore, gaining more insights into T cell exclusion-related lncRNAs could potentially improve understanding the roles of TCE and lncRNAs in immunotherapy.

Currently, there are hardly any studies examining TCE-related lncRNAs in HCC, yet such lncRNAs could be potential therapeutic targets and prognostic markers. In this study, we identified differentially expressed and TCE-associated lncRNAs and used them to develop a prognosis signature to predict immune responses to HCC. The model consisted of 11 lncRNAs and was named 11LNCPS for 11-lncRNA prognosis signature. In addition to OS and TCE, higher 11LNCPS scores had a significant correlation with reduced infiltrations of CD8+ T cells and dendritic cells (DCs) and decreased infiltrations of Th1, Th2, and pro B cells. These infiltration alterations were significantly associated with worse OS in HCC. HCC patients with higher 11LNCPS scores were transcriptomically similar to those who responded better to PDL1 inhibitor. Two of the 11LNCPS lncRNAs, *LINC01134* and *AC116025.2*, were more crucial because their upregulations affected more immune cell types’ infiltrations and were significantly associated with worse OS, TCE, and compromised immune function in HCC. LncRNAs in the 11LNCPS impacted many cancer-associated biological processes and signaling pathways, particularly those involved in immune function and metabolism.

## Materials and Methods

### Data Sources and Processing

**Figure 1:**
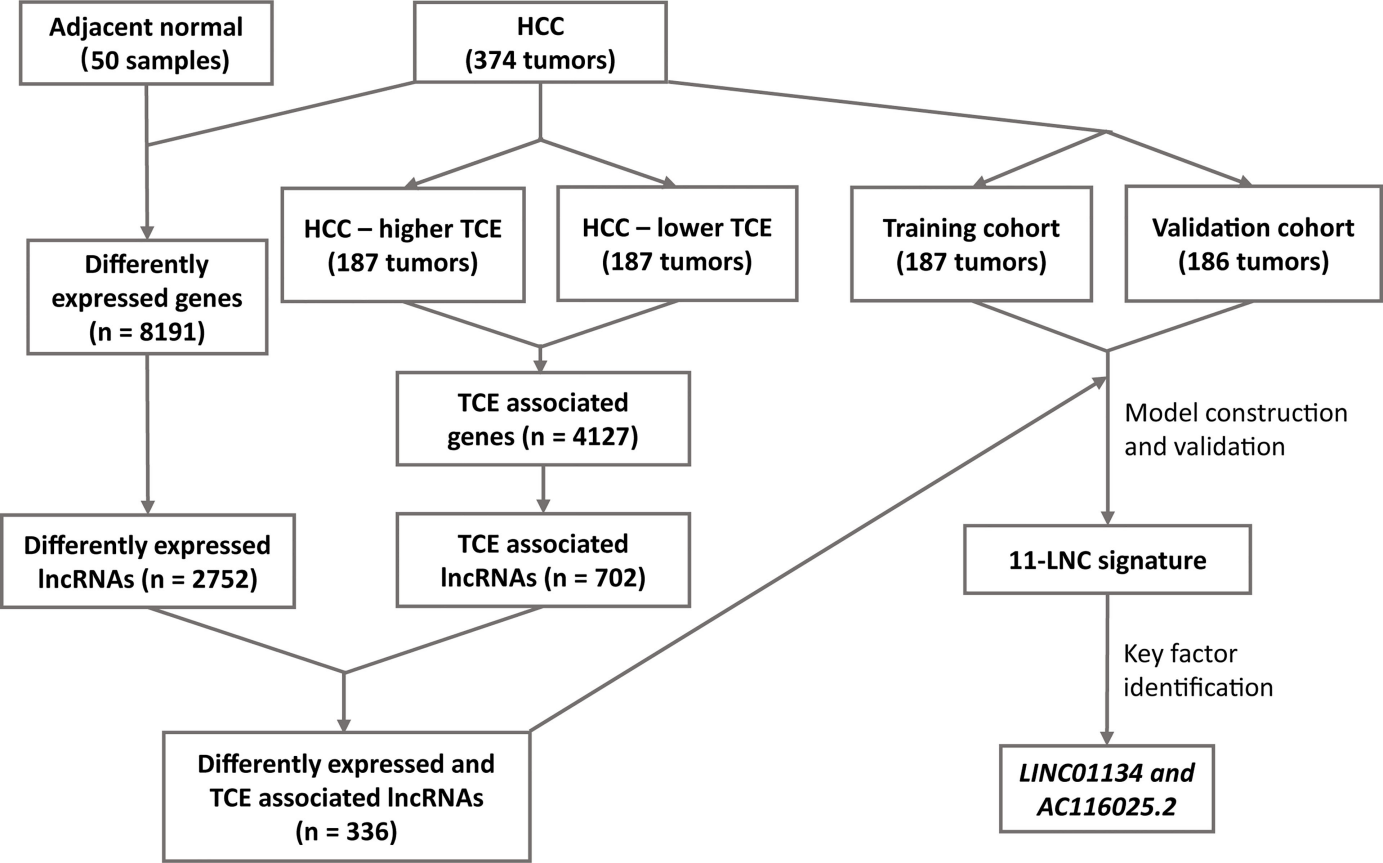
The workflow of the study. Expression data of hepatocellular carcinoma (HCC) and adjacent normal liver tissues were compared to identify differentially expressed lncRNAs in HCC. All HCC with expression data were divided into higher and lower T cell exclusion (TCE) levels using the TIDE analysis. Higher- and lower-TCE groups were compared to identify TCE-associated lncRNAs. Differentially expressed and TCE-associated lncRNAs were then merged to identify differently expressed and TCE-associated lncRNAs, which were then subjected to LASSO and multivariate Cox analyses to construct the 11LNCPS predictive of patient survival. *LINC01134* and *AC116025.2* were then identified as the critical members of the signature.

Gene expression data and clinicopathological characteristics of HCCs used in this study were generated by the Cancer Genome Atlas (TCGA) and are available at https://www.cancer.gov/about-nci/organization/ccg/research/structural-genomics/tcga. Downloaded data included FPKM (fragments per kilobase of transcript per million) reads-based gene expression data and the raw read count values. The R package “TCGAbiolinks” was used for downloading (22). After screening for data quality, 374 HCC samples were retained in this study. Of the 374 cases, one lacked prognostic information, so 373 were used for model construction and survival analyses (**Figure 1**). In addition, the tumor immune dysfunction and exclusion (TIDE) algorithm, as described in a previous study (15), was used to determine both the T cell exclusion (TCE) level and the T cell dysfunction level using the FPKM expression matrix.

To explore what chemokines/cytokines and immune checkpoint ligands mediate the communications between HCC cells and CD8+ T cells, we analyzed a single-cell RNA-sequencing (scRNS-seq) data of HCC (GSE146115) available in the Gene Expression Omnibus (GEO) (23). After performing imputation on the dropouts by the “scImpute” algorithm (24), the R package “Seurat” (25) was used for dimensional reduction, clustering analysis, and cell type annotation. Finally, the CellChat Explorer (26) program was used to infer the biologically significant interactions between chemokines, cytokines, and immune checkpoint (ICP) ligands and their receptors in the interactions between HCC cells and CD8+ T cells.

### Identification of HCC- and TCE-Associated lncRNAs

To identify lncRNAs that are differentially expressed between HCC and adjacent morphologically normal liver tissues, we used the R packages “edgeR” (27, 28) and “limma” (29) to analyze the 374 HCC tissues and 50 adjacent normal liver tissues. The thresholds of *P* ≤ 0.05 and |log2FC| (FC: Fold change) > 0.5 were used. The GENCODE database (30) was used to identify lncRNAs.

After HCC tissues were divided into TCE-higher (n = 187) and TCE-lower (n = 187) groups by the median TCE level following the TIDE analysis, the edgeR-limma procedure was also used to identify differentially expressed lncRNAs between the two TCE groups.

LncRNAs differential expression between HCC and normal tissues and between TCE-higher and TCE-lower groups were identified as HCC- and TCE-associated lncRNAs.

### Construction and Validation of a TCE-Associated lncRNA Prognostic Model: 11LNCPS

The 373 HCCs with prognostic information were randomly assigned to the training cohort (n = 187) and validation cohort (n = 186) at a 1:1 ratio using the R package “caret”. Univariate Cox regression analysis was performed to assess the association of each differentially expressed and TCE-associated lncRNA with the overall survival (OS) in the training cohort. LncRNAs significantly correlated with OS (*P* ≤ 0.001) were subjected to the LASSO-COX regression analysis (31) to develop the prognostic model (i.e., 11LNCPS). Based on the model, a risk score (RS) for OS was built based on a linear combination of the regression coefficient derived from the multivariate Cox regression model and the expression level of the optimized lncRNAs.

The 11LNCPS score (risk score) was computed as follows: $\text{Risk score}=\sum_{i=1}^{N}\left(C_{i}\times Factor_{i}\right)$ , where N represents the number of prognostic factors, Factor_i_ represents the expression of lncRNAs, and C_i_ represents the regression coefficient of the multivariate Cox regression model (1, 32, 33).

HCCs in the training cohort were then divided into two groups using the median, one with higher 11LNCPS scores and the other with lower scores, for model evaluation. Kaplan-Meier analysis was used for overall survival, with the log-rank test to evaluate statistical significance.

The time-dependent receiver‐operating characteristic (ROC) analysis was performed to evaluate the accuracy of the 11LNCPS classifier in survival prediction at 1, 2, and 3 years in the training cohort. The calibration curves, Harrell’s concordance index (C-index) curves, and ROC Areas under the curves (AUCs) were calculated for model evaluation. Calibration curves were calculated by calibrating the function implemented in the “rms” package to assess the predictive ability. The 11LNCPS was also validated in the validation and entire cohorts. In addition, the 11LNCPS model was compared with two previously reported effective HCC prognostic models for ROC, C-index, and prediction error curves in the validation cohort using the packages of “MASS”, “timeROC”, “survival”, and “survminer”. One was the 8-gene model containing *H2AFX*, *SQSTM1*, *ITM2A*, *PFKP*, *TPD52L1*, *ACSL4*, *STRN3*, and *CPEB3* (34); and the other was the 4-gene model containing *CENPA*, *SPP1*, *MAGEB6*, and *HOXD9* (35).

### Analysis of the Association of 11LNCPS Scores With Immune Responses in HCC

Considering that TCE is primarily related to the immune escape (15), we applied the xCell computational method (36–38) to estimate the enrichment scores (xCell scores) of different immune cell types in HCCs with higher and lower 11LNCPS scores.

A total of 374 HCC samples with normalized gene expression FPKM data and standard annotation were used to analyze the distribution of 34 types of immune cells using the xCell pipeline. The 34 types of immune cells included CD4+ naive T-cells, CD4+ T-cells, CD4+ memory T-cells, CD4+ Tcm (central memory T cell), CD4+ Tem (effective memory T cell), CD8+ naive T-cells, CD8+ T-cells, CD8+ Tcm, CD8+ Tcm, Treg cells, gamma delta T cells (Tgd cells), Th1 cells, Th2 cells, natural killer T cell (NKT), natural killer cell (NK), pro B-cells (B cell progenitors), naive B-cells, B-cells, memory B-cells, class-switched memory B-cells, plasma cells, monocytes, macrophages, macrophages M1, macrophages M2, dendritic cell (DC), activated dendritic cell (aDC), conventional dendritic cell (cDC), plasmacytoid dendritic cell (pDC), immature dendritic cell (iDC), neutrophils, eosinophils, mast cells, and basophils.

The Kaplan-Meier (K-M) analysis was performed to assess whether the infiltrating status of different immune cell types affects patient survival in HCC. Survival outcomes were calculated and visualized using the R packages “survival” and “survminer”. The correlation between an xCell score and an immune cell type was analyzed with *P* ≤ 0.05 and |log2 (FC)| > 0.25 following the procedure described previously (36), and the outcome was visualized using the R packages “pheatmap”, “EnhancedVolcano” and “ggpubr”.

TIDE algorithm was then applied to evaluate the association of 11LNCPS scores with TCE and T cell dysfunction. HCCs were divided into higher and lower 11LNCPS scores using the median, and the TIDE algorithm (15) was then applied to each group. The outcome was visualized using “ggpubr”.

To determine whether the 11LNCPS score is associated with therapeutic responses to ICIs, the 373 HCCs were divided into two groups, one with higher and one with lower 11LNCPS scores, using the median. The Subclass Mapping (SubMap) algorithm (39) was then applied to measure the correspondence between the two 11LNCPS groups and groups of malignancies with and without responses to anti‐CTAL‐4, anti‐PD‐1, and anti‐PD‐L1 therapies from previous studies (40, 41). The outcome was visualized using the R packages of “pheatmap” and “ggpubr”.

### Gene Ontology (GO) Enrichment, Kyoto Encyclopedia of Genes and Genomes (KEGG) Pathway Analysis, and Gene Set Enrichment Analysis (GSEA)

To determine the critical biological pathways and characteristics of HCC determined by 11LNCPS score, the GO, KEGG pathway analysis, and GSEA (42, 43) were applied to HCCs with higher and lower 11LNCPS scores using the R packages “GSEABase”, “clusterProfiler” (44), “enrichplot”, and “org.Hs.eg.db”. Briefly, the edgeR-limma procedure was used to find differentially expressed genes (DEGs) between HCCs with higher-RS and those with lower RS in TCGA. DEGs with thresholds of *P* ≤ 0.05 and |log2FC| (FC: Fold change) > 0.5, were subjected to GO and KEGG analysis. Go analysis included BP (biological process), CC (cellular component), and MF (molecular function). For GSEA, all DEGs were subjected to the “GSVA” package (45) after ranking from high to low based on their FC values. A *P* value smaller than 0.05 was considered significant in the GSEA. The hallmark gene set “h.all.v7.1.symbols.gmt” was downloaded from https://www.gsea-msigdb.org/and subjected to “GSVA” in a similar fashion.

### Identification of Critical Members of the 11LNCPS lncRNAs

For each of the 11 lncRNAs in the 11LNCPS, a series of analyses were performed to identify the core one. The Kaplan-Meier survival analysis was performed to assess whether a lncRNA’s expression level is associated with the overall survival (OS) in the 373 HCC patients.

Correlation of each lncRNA’s expression level with T cell exclusion (TCE) was ranked based on the Spearman correlation coefficient value, with those greater than 0.2 with *P* ≤ 0.05 considered significant. Infiltration levels of prognosis-associated immune cells were also compared between HCCs with higher and lower 11LNCPS scores and HCCs with higher and lower expression levels of the 11LNCPS lncRNAs. Those with a lower level. LncRNAs whose higher expression levels significantly correlated with worse patient OS, whose Spearman correlation coefficient values were greater than 0.2 (*P* ≤ 0.05), and that affected infiltrations of more immune cell types were considered crucial members of the 11LNCPS, including *LINC01134* and *AC116025.2*. The outcome was visualized by the R packages “pheatmap”, “ggpubr”, “corrplot” and “ggplot2”.

### Test of Whether *LINC01134* and *AC116025.2* Affect TCE and T Cell Dysfunction

The relationship between *LINC01134* and *AC116025.2* expression and TCE or T cell dysfunction was tested using the TIDE algorithm, and the outcome was visualized using the “ggpubr” R package.

### Enrichment Analysis for Biological Functions Affected by the Critical 11LNCPS lncRNAs

To explore the biological functions of *LINC01134* and *AC116025.2* in HCC, we performed GO, KEGG, and GSEA analysis in HCCs as described in the previous enrichment analysis.

### Cell Lines and Cell Culture

Normal liver cells QSG-7701 and LO2 were kindly provided by Dr. Liang Yang of the Southern University of Science and Technology. HCC cell lines HepG2 and Huh-7 were purchased from the BeNa Culture Collection (Beijing, China). The Jurkat cell line was kindly provided by Dr. Lili Ren of Shenzhen People’s Hospital. The DMEM medium (Gibco, USA) supplemented with antibiotics (Biological Industries, Israel) and 10% FBS (Gibco) was used for liver cell culture. The RPMI 1640 medium (Gibco) supplemented with antibiotics and 10% FBS were used for Jurkat cells. All cells were cultured at 37 °C in a humidified atmosphere containing 5% CO_2_.

### Cell Transfection and Conditioned Medium (CM) Preparation

Both the negative control siRNAs (si-NC) and the *LINC01134* siRNAs were provided by GenePharma (Shanghai, China). Sequences of siRNAs against *LINC01134* were 5′-GACAGGTTTGAGCTAGAAAC-3′ (si-*LINC01134*-1) and 5′-GCAAAUGCACAGCGAGGAAAG-3′ (si-*LINC01134*-5). At confluency of 30–50%, HepG2 or Huh-7 cells were transfected with siRNAs using the Lipofectamine RNAiMAX reagent (Invitrogen, USA). After 48 hours, transfected cells were split into two portions. One was used for RNA isolation and gene expression analysis, and the other was grown in a 6-well plate for 48 hours to collect the conditioned medium (CM). Each experiment was repeated twice unless otherwise stated.

### Quantitative Real-Time Polymerase Chain Reaction (qRT-PCR)

Total RNA was extracted from cultured cells using the Eastep Super Total RNA Extraction Kit (Promega, USA) and reverse transcribed into cDNA using the HiScript III All-in-one RT SuperMix Perfect for qPCR Kit (Vazyme, China). PCR was performed with the KT SYBR qPCR Mix (Ktsm-life, China) using the qTOWER 3.0 PCR system (Jena Industries, Germany). Primers and their sequences are as follow: *LINC01134*, 5′-ATGAACAGCAAATGCACAGCG-3′ (forward) and 5′- ATAGGTCTTGGCTGGTTCTCG-3′ (reverse); *AC116025.2*, 5′-TGGAGCAGAAAGAGCTGTCTCAAG-3′ (forward) and 5′-TGTCAGGAAACTGTGTGGACG-3′ (reverse); *CXCL1*, 5′-CTGGCTTAGAACAAAGGGGCT-3′ (forward) and 5′-TAAAGGTAGCCCTTGTTTCCCC-3′ (reverse); *CXCL2*, 5′-CCCATGGTTAAGAAAATCATCG-3′ (forward) and 5′-CTTCAGGAACAGCCACCAAT-3′ (reverse); *CXCL3*, 5′-CGCCCAAACCGAAGTCATAG-3′ (forward) and 5′-ACCTTGCCTTCTTTGTCTTTGTTGGA-3′ (reverse); and β-actin, 5′-TCCCTGGAGAAGAGCTACGA-3′ (forward) and 5′-GCTCCCCTTGTTCAGTATCTTTT-3′ (reverse). In the PCR, β-actin served as the endogenous control. The relative expression of genes was calculated using the −2ΔΔCt method.

### T Cell Migration Analysis

T cell migration was analyzed using the transwell assay as previously described (46–48). Briefly, Jurkat cells (10^6^ cells/ml) were washed with PBS and serum-starved for 3 hours, 10^5^ cells in 0.1 ml were then seeded onto an 8.0-μm pore size insert (Corning, USA), and 400 μl complete medium or CM were then added to the lower chambers of a 24-well plate (Corning). After incubation at 37°C for 16 hours in an incubator, migrated cells in the lower chambers were collected and counted using an automated cell counter (Invitrogen). The numbers of migrated cells in different groups were normalized by the number of cells from the complete medium group.

### Statistical Analysis

The R software (version 4.1.1) was used for all statistical analyses and plot drawings except as specifically stated. Patients were randomly grouped using the “caret” R package. The univariate and multivariate Cox proportional hazards regression analyses were performed using the “survival” package. Kaplan-Meier analysis was used for overall survival, with the log-rank test to evaluate statistical significance. Statistical differences between the two groups were assessed using the Wilcoxon test. The grouping basis (the cutoff point) was the median value of each corresponding index.One-way ANOVA with Bonferroni’s multiple-comparisons test was performed for qPCR and T cell migration analysis using the GraphPad Prism (GraphPad Prism 8). *P* < 0.05 was considered statistically significant unless otherwise stated.

## Results

### Identification of Differentially Expressed and TCE-Associated lncRNAs in HCC

**Figure 2:**
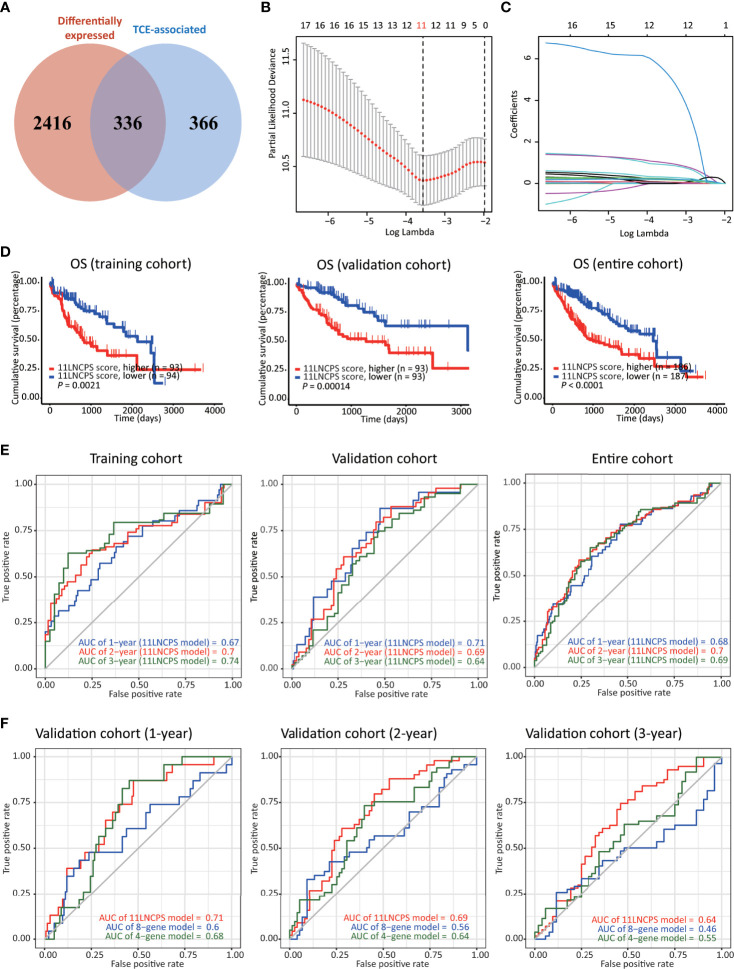
Construction, validation, and evaluation of an 11-lncRNA signature predictive of prognosis (11LNCPS) in HCC patients. **(A)** Venn diagram showing the overlapping lncRNAs (n = 336) between lncRNAs differentially expressed in HCC (n = 2752, red) and those associated with T cell exclusion (TCE, n = 702, blue). **(B)** Partial likelihood deviance of varying numbers of prognostic lncRNAs revealed by the LASSO regression model. The grey lines represent the partial likelihood deviance ± standard error (SE). The two vertical lines represent optimal values based on the minimum criteria and 1-SE criteria. The proper log (Lambda) value was chosen *via* the minimum criteria. **(C)** Identification of 11 lncRNAs by the LASSO logistic regression model with non-zero coefficients. **(D)** The Kaplan–Meier analysis of overall survival (OS) in the training cohort (left), validation cohort (center), and entire cohort (right) cohort of TCGA HCC patients with higher and lower 11LNCPS scores based on the median. The cutoff value of group dividing was the median RS score. **(E)** Receiver operating characteristic (ROC) curves of the 11LNCPS model for evaluating the predictability of OS in 1, 2, and 3 years in the training cohort (left), validation cohort (center), and entire cohort (right) cohort. **(F)** Comparison of ROC curves between the 11LNCPS model (red) and the previously established 8-gene model (blue) and 4-gene model (green) for 1, 2, and 3 years OS in the validation cohort.

The workflow of the entire study is summarized in **Figure 1**. In total, the TCGA database contained 374 HCC cases with gene expression data. All the 374 cases were used for the identification of differentially expressed and TCE-associated lncRNAs. One of the 374 cases lacked prognostic information and thus was excluded for model construction and survival analysis. Using the 374 HCCs and 50 cases of noncancerous liver tissues with expression profiling and other information, two groups of differentially expressed genes (DEGs) were identified from a total of 56493 human genes, including lncRNA, other noncoding RNA, and protein coding genes. One group contained 8191 genes that were differentially expressed between HCCs and normal liver tissues, with 6438 upregulated and 1753 downregulated in HCC (**Figure 1** and **Figure S1A**). Among these 8191 DEGs, 2752 were lncRNAs (**Figure 1**). The other group contained 4127 TCE-associated genes that were differentially expressed between HCCs with higher TCE scores and those with lower TCE scores, including 2914 upregulated and 1213 downregulated in the TCE-higher group (**Figure 1** and **Figure S1B**). Of the 4127 TCE-associated genes, 702 were lncRNAs (**Figure 1**). In total, 336 lncRNAs were both differentially expressed and TCE-associated in HCC (**Figure 2A** and **Table S1**).

### Construction of the TCE-Associated 11 lncRNA Prognostic Signature (11LNCPS) in HCC

Of the 374 HCC cases, one lacked prognostic information and thus was excluded for model construction and survival analysis. The 373 HCCs with survival data were divided into the training (n = 187) and validation (n = 186) cohorts. Each differentially expressed TCE-associated lncRNA in the training cohort was subjected to the univariate Cox regression analysis to evaluate its association with patients’ overall survival (OS). Fifty-four lncRNAs were significantly associated with prognosis (*P* < 0.001) (**Table S1**). The LASSO-Cox regression analysis was then performed, in which tenfold cross-validation was applied to overcome overfitting with an optimal λ value of 0.028393 selected (**Figure 2B**). A combination of 11 lncRNAs had non-zero LASSO coefficients and thus was the most robust prognostic value (**Figure 2C**). This combination of 11 lncRNAs was named 11 lncRNA prognostic signature (11LNCPS). The 11 lncRNAs included *LINC01134*, *C2orf27A*, *LINC00501*, *AC104066.3*, *AC034229.4*, *CASC8*, *FAM225B*, *AL451069.3*, *AL161669.3*, *AC116025.2* and *LINC00632*.

To determine the 11LNCPS score, the Cox multivariate regression analysis was used to evaluate each of the 11 lncRNA’s contribution to the 11LNCPS (**Table 1**), which resulted in the following formular for calculating the risk score (i.e., 11LNCPS score) in an HCC: 11LNCPS score = 0.214579 × expression of *LINC01134* + 0.019508 × expression of *C2orf27A* + 1.045738 × expression of *LINC00501* + 1.244493 × expression of *AC104066.3* + 0.140677 × expression of *AC034229.4* + 0.302498 × expression of *CASC8* + 7.231505 × expression of *FAM225B* + 0.089521 × expression of *AL451069.3* + 0.226717 × expression of *AL161669.3* + 0.224378 × expression of *AC116025.2* + 0.371662 × expression of *LINC00632*.

**Table 1:** Univariate and multivariate Cox regression analysis for overall survival in the training cohort of HCCs from TCGA (n = 187).

Variables	Univariate analysis				Multivariate analysis			
	HR	*P* value	HR.95L	HR.95H	HR	*P* value	HR.95L	HR.95H
*LINC01134*	2.957099	**0.001928**	1.490262	5.867717	1.23934	0.667749	1.49026198	5.8677175
*C2orf27A*	1.713252	**0.000121**	1.301973	2.254449	1.0197	0.932463	1.30197261	2.25444941
*LINC00501*	5.639399	**3.96E-05**	2.47158	12.8674	2.845499	0.124401	2.47158045	12.8674025
*AC104066.3*	7.467815	**0.003104**	1.969993	28.30886	3.471175	0.139087	1.96999337	28.3088586
*AC034229.4*	1.903967	**0.003121**	1.242232	2.918208	1.151052	0.67269	1.24223153	2.91820769
*CASC8*	1.554184	**0.00019**	1.232892	1.959205	1.353236	**0.043159**	1.23289183	1.95920478
*FAM225B*	7935.61	**0.008989**	9.418916	6685897	1382.301	0.108321	9.41891643	6685896.61
*AL451069.3*	1.115362	**0.009697**	1.026798	1.211566	1.093651	0.101831	1.02679797	1.21156582
*AL161669.3*	1.250841	**0.000155**	1.113895	1.404623	1.254475	**0.001296**	1.1138952	1.40462346
*AC116025.2*	2.414076	**0.000745**	1.446456	4.028994	1.251545	0.526933	1.44645621	4.02899437
*LINC00632*	1.833973	**0.005234**	1.198149	2.807209	1.450143	0.153236	1.19814937	2.80720913

HR, hazard ratio; HR.95L, low 95% confidence interval of HR; HR.95H, high 95% confidence interval of HR. Significant P values (≤ 0.05) are in bold.

### Construction of the TCE-Associated 11 lncRNA Prognostic Signature (11LNCPS) in HCC

To test the validity and effectiveness of the 11LNCPS in HCC, we calculated the 11LNCPS risk score for each case in the training, validation, and entire cohorts; divided HCCs in each cohort into the higher- and lower-risk groups using the median 11LNCPS score; and performed a series of analyses (**Figures 2D–F** and **Figures S2A–D**). The Kaplan-Meier analysis demonstrated that the OS rate was better in patients with lower 11LNCPS scores than those with higher scores in each cohort (*P* ≤ 0.05, **Figure 2D**).

The area under ROC curve (AUC) for 1, 2, and 3 years reached 0.67, 0.7, and 0.74, respectively, in the training cohort; 0.71, 0.69, and 0.64, respectively, in the validation cohort; and 0.68, 0.7, and 0.69, respectively, in the entire cohort (**Figure 2E**). These AUC curves indicate a reasonable discrimination power of the 11LNCPS in HCC. Additionally, the 11LNCPS’s C-index was greater than 0.60 for 1, 2, and 3 years in each cohort, showing an excellent predictive accuracy of the 11LNCPS (**Figure S2A**). Furthermore, the calibration curve demonstrated good consistency for 1, 2, and 3 years in each cohort (**Figure S2B**).

We also compared our 11LNCPS model with two reported models, i.e., the 8-gene model (34) and the 4-gene model (35) in the validation cohort. For each of the 3 time points (1, 2, and 3 years), 11LNCPS showed a higher AUC value (**Figure 2F**) and a higher C-index (**Figure S2C**). Each model’s predicted error line overlapped well with the reference line (**Figure S2D**), demonstrating a lower predicted error rate for each of the 3 models.

### The 11LNCPS Scores Nicely Correlate With Immune Responses to HCC

**Figure 3:**
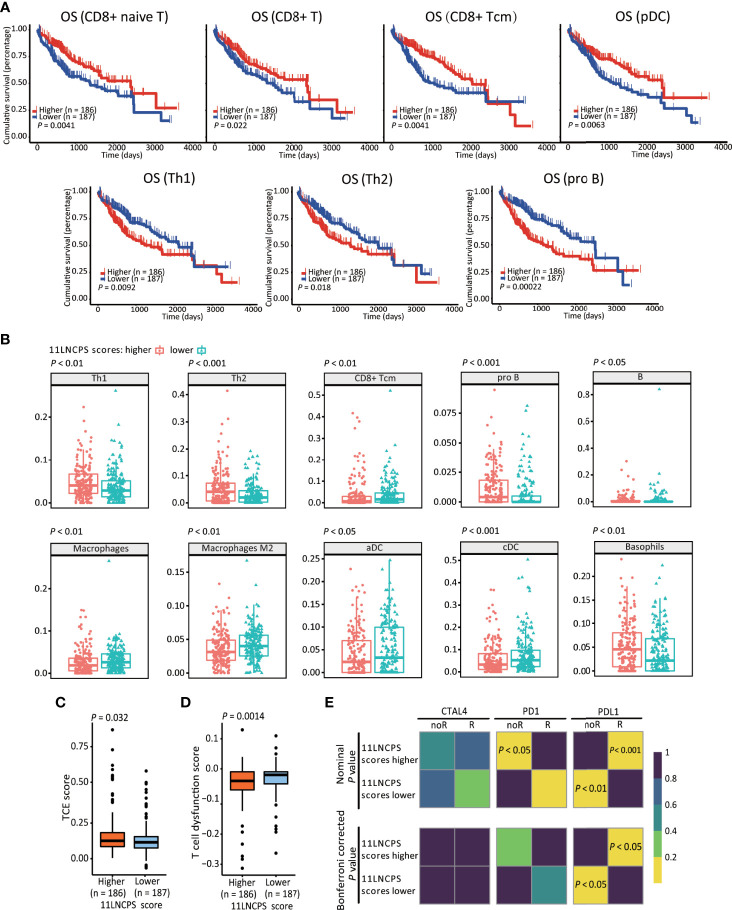
The 11LNCPS scores predict immune responses in HCC. **(A)** Increased infiltrations of Th1, Th2, and pro B cells are associated with worse OS, while that of CD8+ Tcm, CD8+ T, and pDC cells with better OS in HCC, as determined by the Kaplan-Meier analysis. **(B)** The infiltration level is different (*P* < 0.05) between HCCs with higher 11LNCPS scores (red) and lower scores (blue) for 10 types of immune cells. **(C, D)** HCCs with higher 11LNCPS scores have higher TCE scores **(C)** and lower T cell dysfunction scores **(D)**. **(E)** Higher 11LNCPS scores are associated with better therapeutic responses to immune checkpoint inhibitors (ICIs) in HCC patients. Nominal and Bonferroni corrected *P* values are shown for the correlation between 11LNCPS scores and ICI responses (CTAL4, PD1, and PD-L1). noR, non-responder; R, responder. Grid colors indicate the correlation *P* values.

We applied the xCell algorithm to the RNA-seq datasets of the 374 HCCs to determine the infiltration levels of 34 types of immune cells (**Figure S3**). The correlation between an immune cell infiltration and patient OS was evaluated using the Kaplan-Meier analysis (**Figure 3A** and **Figure S4**). Altered infiltrations of 7 types of immune cells were significantly associated with OS (**Figure 3**). Increased infiltrations of CD8+ naive, CD8+ Tcm, CD8+ T, and pDC cells were associated with better OS, while increased infiltrations of Th1, Th2, and pro B cells were associated with a worse OS in HCC (**Figure 3A**).

To evaluate the relationship between the 11LNCPS and immune responses to HCC, we divided all HCCs into higher and lower 11LNCPS scores using the median and compared the distribution of different immune cell types between the two groups (**Figure 3B** and **Figure S5**). HCCs with higher 11LNCPS scores had decreased infiltrations of CD8+ Tcm, macrophages, macrophages M2, aDCs, and cDCs immune cells and increased infiltrations of Th1, Th2, pro B, B, and basophils cells (**Figure 3B**). In the Kaplan-Meier analysis, alterations in 4 of the 10 immune cell types were significantly associatged with OS (**Figure 3A**). The 4 alterations included decreased filtration of CD8+ Tcm cells and increased filtrations of Th1, Th2, and pro B cells (**Figures 3A, B**).

TIDE is a computer program that models the induction of T cell dysfunction in tumors with higher infiltration of cytotoxic T cells and the prevention of T cell infiltration in tumors with lower levels of such cells (15). To further explore the impact of 11LNCPS lncRNAs on immune responses in HCC, we compared HCCs with higher and lower 11LNCPS scores for TCE and T cell dysfunction levels which were analyzed using the TIDE program. HCCs with higher 11LNCPS scores had significantly higher TCE scores and lower T cell dysfunction levels than those with lower 11LNCPS scores (*P* ≤ 0.05) (**Figures 3C, D**).

Using the SubMap analysis, we compared HCCs with higher and lower 11LNCPS scores to malignancies with and without responses to immunotherapies from previous studies (41, 49). HCCs with higher 11LNCPS scores were significantly associated with malignancies that respond to a PDL1 inhibitor (**Figure 3E**, *P* < 0.05).

### Functional Impact of the 11LNCPS on HCC Cells

**Figure 4:**
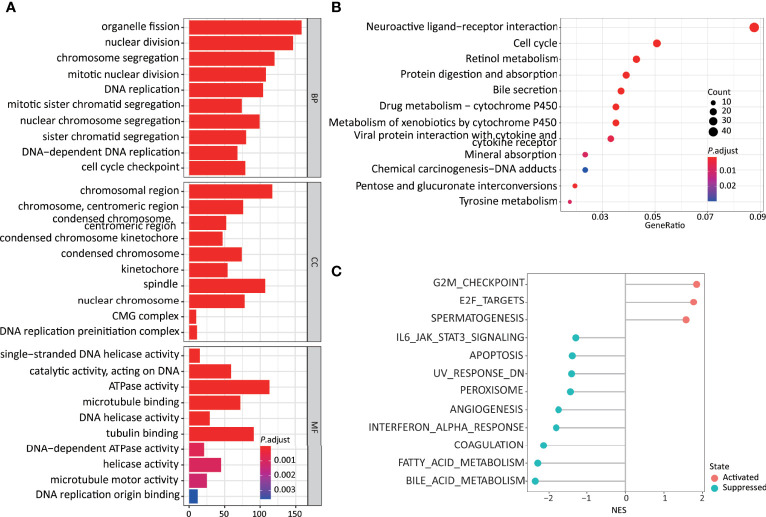
Higher 11LNCPS scores are associated with several cancer hallmarks and immunological characteristics of HCC. **(A, B)** GO enrichment **(A)** and KEGG pathway **(B)** analysis of differentially expressed genes (DEGs) between HCCs with higher and lower 11LNCPS scores. The heights of bars and sizes of dots represent the count of genes, while the colors represent the adjusted *P*-value. **(C)** Significantly enriched cancer hallmarks in HCCs with higher 11LNCPS scores, as analyzed by the GSEA. Red and blue dots indicate a pathway’s activation and suppression, respectively. The x-axis shows normalized enrichment scores (NES). All pathways with *P* values smaller than 0.05 are shown.

Differentially expressed genes were identified in HCCs with higher and lower 11LNCPS scores (**Figure S6**). Such genes were analyzed to evaluate the effect of 11LNCPS lncRNAs on different biological processes and signaling pathways using the GO, KEGG pathway, and GSEA analyses (**Figure 4**).

Many biological processes identified in the GO analysis are involved in the cell cycle and DNA replication. These processes included organelle fission, nuclear division, DNA-dependent DNA replication, cell cycle checkpoint, chromosomal region, DNA replication preinitiation complex, single-stranded DNA helicase activity, ATPase activity, and DNA replication origin binding.

In the KEGG pathway analysis, the top-ranked pathways were involved in cell cycle and ligand-receptor interactions, including cytokine and cytokine receptor-related signaling and the viral proteins’ interactions with cytokines and cytokine receptors (**Figure 4B**). They also included metabolism-associated pathways such as retinol, drug, and xenobiotics (**Figure 4B**).

The GSEA analysis resulted in similar findings (**Figure 4C**). Specifically, signaling pathways related to cell cycle and DNA replication were significantly enriched in HCCs with higher 11LNCPS scores, including G2M checkpoint, E2F targets, cell cycle, and UV response containing DNA replication genes. Signaling pathways related to metabolism, immune function, and cell death were significantly suppressed in HCCs with higher 11LNCPS scores, including fatty acid metabolism, bile acid metabolism, IL6-JAK-STAT3 signaling, IFNα response, and apoptosis.

Therefore, the 11LNCPS appears to affect cell cycle signaling pathways, DNA replication, immune function, and cell death.

### *LINC01134* and *AC116025.2* Are Most Crucial Than Other lncRNAs in the 11LNCPS

**Figure 5:**
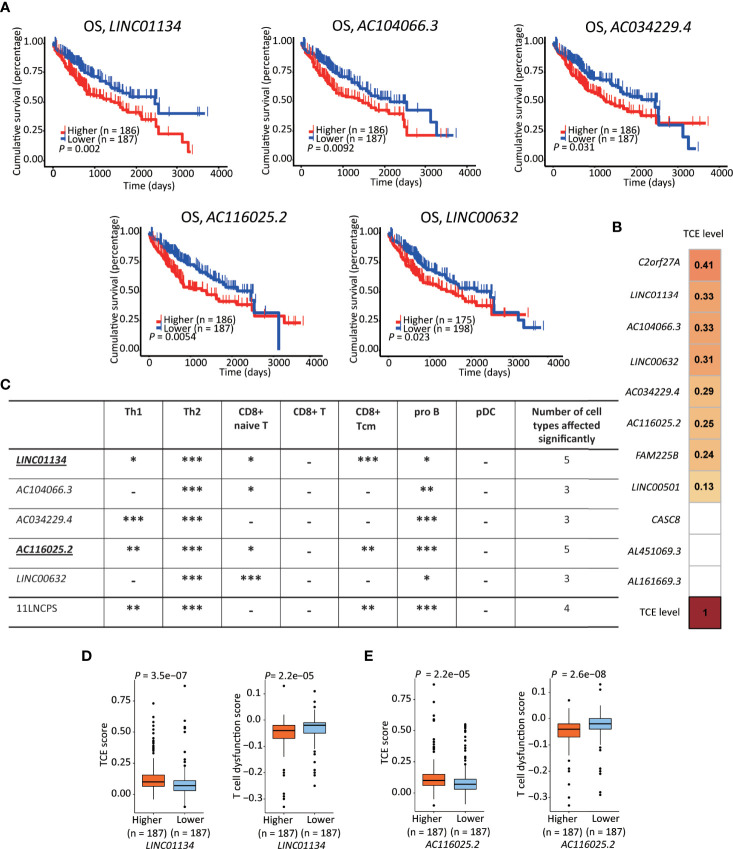
*LINC01134* and *AC116025.2* are the most crucial lncRNAs of the 11LNSPS. **(A)** An association of higher expression level with worse OS in HCC patients was detected for 5 of the 11LNCPS lncRNAs, including *LINC01134*, *AC104066.3*, *AC034229.4*, *AC116025.2*, and *LINC00632*, as determined by the Kaplan-Meier survival analysis. **(B)** Coefficient values for each lncRNA in the 11LNCPS, as indicated in colored grids and determined by the Spearman analysis. Colored grids indicate those whole expression alterations were statistically significant. **(C)** Statistical evaluation of the correlation between the infiltration (indicated by an xCell score) of a prognosis-associated immune cell type and expression levels of prognosis-associated lncRNAs in HCC. HCCs were divided into higher and lower groups using its median expression level for each lncRNA, and xCell scores for each immune cell type were compared between the two groups by the Wilcoxon test. The 11LNCPS was used as a control. -*P* > 0.05; *P ≤ 0.05; **P ≤ 0.01; ***P ≤ 0.001. **(D, E)** Higher *LINC01134* **(D)** and *AC116025.2* **(E)** levels are associated with higher TCE scores and reduced T cell dysfunction levels in HCC, as analyzed by the TIDE algorithm.

To rank the 11LNCPS’s 11 lncRNAs for their contributions to the signature, we analyzed them for the association of expression change with OS and immune responses in HCC. In the Kaplan-Meier analysis, the increased expression in 5 of the 11 lncRNAs was significantly associated with worse OS, including *LINC01134*, *AC104066.3*, *AC034229.4*, *AC116025.2*, and *LINC00632* (**Figure 5A** and **Figure S7**).

Based on the TCE scores revealed by the Spearman analysis, increased expression in 8 of the 11 lncRNAs was significantly associated with TCE (*P* < 0.05). These 8 lncRNAs and their Spearman coefficient values were *C2orf27A*, 0.41; *LINC01134*, 0.33; *AC104066.3*, 0.33; *LINC00632*, 0.31; *AC034229.4*, 0.29; *AC116025.2*, 0.26; *FAM225B*, 0.24; and *LINC00501*, 0.13, respectively (**Figure 5B**).

We further evaluate their effects on immune cell infiltration for the 5 lncRNAs whose expression increase was significantly associated with a worse OS.

While expression change in *LINC01134* or *AC116025.2* significantly affected the infiltrations of 5 immune cell types, expression change in other 11LNCPS lncRNAs altered 3 or 4 types (**Figure 5C** and **Figure S8**). Specifically, *LINC01134* and *AC116025.2* upregulation was significantly associated with increased infiltrations of Th1, Th2, and pro B immune cells but decreased infiltrations of CD8+ naive T and CD8+ Tcm cells (**Figure 5C** and **Figures S8A, D**). For the other 3 11LNCPS lncRNAs associated with OS, *AC034229.4* upregulation was associated with increased infiltrations of Th1, Th2, and pro B cells (**Figure S8C**); and higher levels of *AC104066.3* and *LINC00632* were associated with increased infiltrations of Th2 and pro B cells and decreasing infiltration of CD8+ naive T cells (**Figures S8B, E**).

Additionally, HCCs with higher *LINC01134* or *AC116025.2* expression had higher TCE scores and reduced T cell dysfunction levels (**Figures 5D, E**).

### Upregulation of *LINC01134* and *AC116025.2* Could Impact Immune Responses and Other Biological Processes in HCC

Similar to the analyses of 11LNCPS for its potential impact on biological processes and signaling pathways, we divided HCCs with higher and lower expression levels of *LINC01134* or *AC116025.2*, identified differentially expressed genes, and performed GO, KEGG pathway, and GSEA analyses (**Figure S9**).

**Figure 6:**
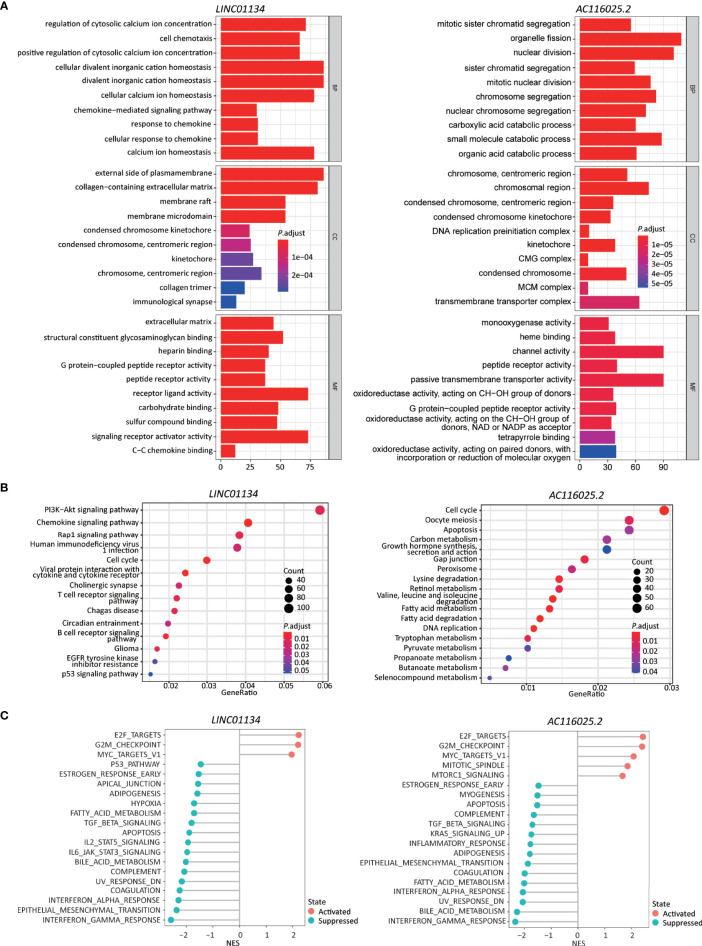
Higher expression levels of *LINC01134* and *AC116025.2* are associated with several cancer hallmarks and immunological characteristics of HCC. **(A, B)** GO enrichment **(A)** and KEGG pathway **(B)** analyses between HCCs with higher- and lower-levels of *LINC01134* (left in each panel) and *AC116025.2* (right in each panel). The heights of bars and sizes of dots represent the count of genes, while the colors represent the adjusted *P*-value. **(C)** Significantly enriched cancer hallmarks in HCCs with higher expression levels of *LINC01134* (left) and *AC116025.2* (right), as analyzed by the GSEA. The red and blue colors of dots indicate a pathway’s activation and suppression, respectively. The x-axis shows normalized enrichment scores (NES). All pathways with *P* values smaller than 0.05 are shown.

The most enriched processes for *LINC01134* in the GO enrichment analysis included cell chemotaxis and chemokine response related biological processes, chromosome related molecular function, receptor-ligand activity, and chemokine binding cellular component (**Figure 6A**, left).

In the KEGG pathway analysis, *LINC01134* upregulation was significantly associated with diverse immune-related signaling pathways, including chemokines/cytokines and their receptors and T and B cell receptors. Some cancer-associated pathways were identified, including PI3K-Akt, Rap1, cell cycle, glioma, and p53 (**Figure 6B**, left).

In the GSEA analysis, *LINC01134* upregulation was associated with the active cell cycle (e.g., E2F targets and G2M checkpoint). It was also associated with cancer pathways (e.g., MYC targets) (**Figure 6C**, left). On the other hand, *LINC01134* upregulation was inversely related to pathways of immune (IFNγ response, IFNα response, IL6-JAK-STAT3, IL2-STAT5), metabolism (bile acid metabolism, fatty acid metabolism), apoptosis, epithelial-mesenchymal transition, UV response, TGFβ, hypoxia, and p53 (**Figure 6C**, left).

These results support the role of *LINC01134* in the cell cycle, cell death, immunity, chemokine expression, and chemotaxis in HCC.

In the GO analysis, *AC116025.2*-associated genes were primarily enriched in cell division, catabolic metabolism, chromosome and transporter complex, receptor and channel activities, and oxidoreductase activity (**Figure 6A**, right).

In the KEGG analysis, *AC116025.2*-associated genes were enriched for pathways in the cell cycle, DNA replication, metabolism, and apoptosis (**Figure 6B**, right). Multiple metabolic pathways were enriched, including carbon metabolism, retinol metabolism, fatty acid metabolism, tryptophan metabolism, and propanoate metabolism (**Figure 6B**, right).

In the GSEA, *AC116025.2* upregulation was associated with cell cycle activities and cancer-related pathways such as E2F targets, G2M checkpoint, MYC targets, MTORC1 signaling, and mitotic spindle (**Figure 6C**, right). On the other hand, *AC116025.2* upregulation was associated with reduced activities of signaling pathways related to immune, metabolism, and cell death, including IFNγ response, IFNα response, inflammatory response, bile acid metabolism, fatty acid metabolism, apoptosis, UV response, epithelial-mesenchymal transition, KRAS signaling, TGFβ signaling, and estrogen response (**Figure 6C**, right).

### *LINC01134* and *AC116025.2* Upregulation Correlates With the Expression of Some Chemokines, Cytokines, and ICP Ligands

Immune responses often involve cytokines, chemokines, and their receptors. Therefore, we investigated whether expression changes in *LINC01134* and *AC116025.2* are associated with chemokines, cytokines, and ICP ligands in HCC. In the scRNA-seq data, CD8+ cells could be annotated (**Figure S10A**). We thus identified the chemokines, cytokines, and ICP ligands synthesized by HCC cells and could mediate CD8+ T cells’ recruitment using the CellChat algorithm (26).

**Figure 7:**
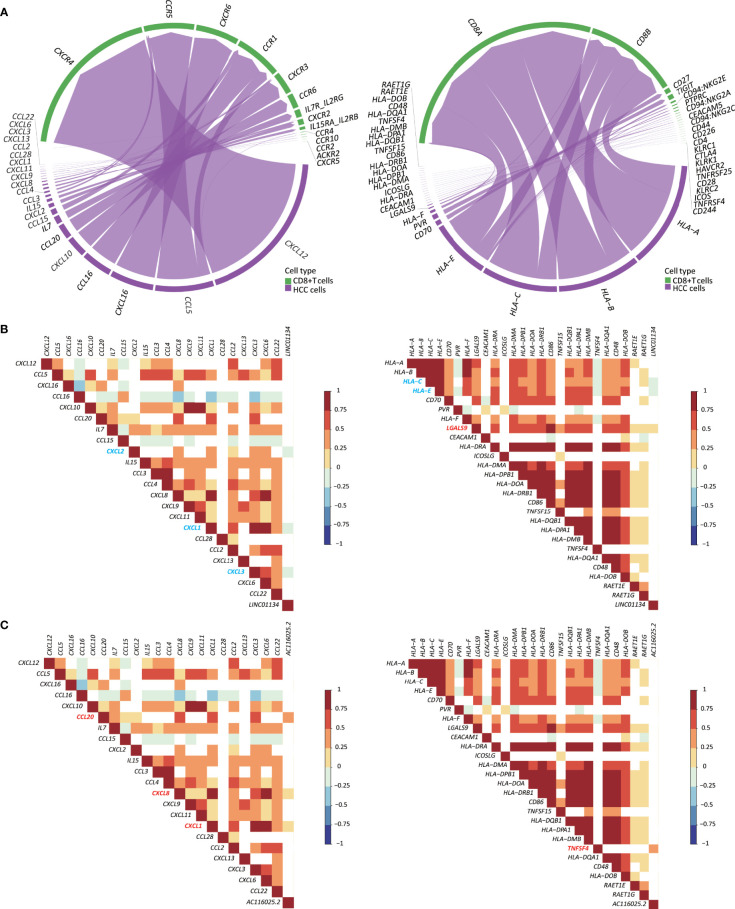
Expression of *LINC01134* and *AC116025.2* is associated with the expression of some cytokines, chemokines, and immune checkpoint (ICP) ligands in HCC. **(A)** The chord diagram shows heterotypic signal transduction between HCC cells (purple) and CD8+ T cells (green), with purple arrows pointing from cytokines and chemokines (left) or ICP ligands (right) in HCC cells to their respective receptors in CD8+ T cells. **(B, C)** Expression of *LINC01134* **(B)** and *AC116025.2* **(C)** is associated with the expression of some cytokines and chemokines (left) or ICP ligands (right), as determined by the Spearman analysis. Grid colors and gradient color bars indicate Spearman coefficient values, with white color indicating a lack of statistical significance. Cytokines, chemokines, and ICP ligands with a positive association with *LINC01134* or *AC116025.2* expression are marked by red, while those with a negative correlation are marked by blue.

In total, 22 cytokines and chemokines were identified, including *CXCL12*, *CCL5*, *CXCL16*, *CCL16*, *CXCL10*, *CCL20*, *IL7*, *CCL15*, *CXCL2*, *IL15*, *CCL3*, *CCL4*, *CXCL8*, *CXCL9*, *CXCL11*, *CXCL1*, *CCL28*, *CCL2*, *CXCL13*, *CXCL3*, *CXCL6*, and *CCL22* (**Figure 7A**, left). We also identified 26 ICP ligands that could bind to their ICPs, including *HLA-A*, *HLA-B*, *HLA-C*, *HLA-E*, *CD70*, *PVR*, *HLA-F*, *LGALS9*, *CEACAM1*, *HLA-DRA*, *ICOSLG*, *HLA-DMA*, *HLA-DPB1*, *HLA-DOA*, *HLA-DRB1*, *CD86*, *TNFSF15*, *HLA-DQB1*, *HLA-DPA1*, *HLA-DMB*, *TNFSF4*, *HLA-DQA1*, *CD48*, *HLA-DOB*, *RAET1E*, and *RAET1G* (**Figure 7A**, right). Using the Spearman correlation analysis, we found that *LINC01134* upregulation in HCC was negatively correlated with the following genes (R_S_ > 0, *P* ≤ 0.05): *CXCL1*, *CXCL2*, *CXCL3*, *HLA-C*, and *HLA-E* and was positively correlated with *LGALS9* (R_S_ < 0, *P* ≤ 0.05) (**Figure 7B**). For *AC116025.2*, its upregulation was positively associated with *CXCL1*, *CXCL8*, *CXCL20*, and *TNFSF4* (R_S_ > 0, *P* ≤ 0.05) (**Figure 7C**).

### Upregulation of *LINC01134* and *AC116025.2* in HCC Cell Lines and the Impact of *LINC01134* on *CXCL2* and *CXCL3* Expression and T Cell Migration

**Figure 8:**
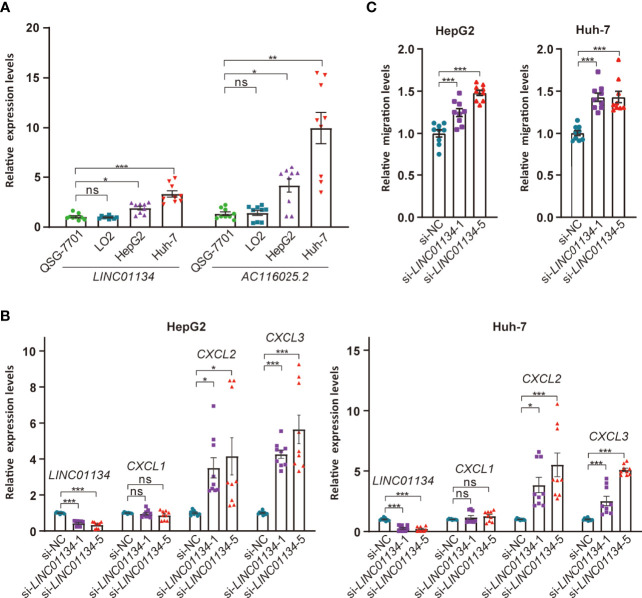
Expression and functional tests of key member lncRNAs of the 11LNCPS in HCC cell lines. **(A)** Expression of *LINC01134* and *AC116025.2* in normal liver cell lines QSG-7701 and LO2 and HCC cell lines HepG2 and Huh-7, as detected by qRT-PCR. Data were normalized by β-actin mRNA levels and standardized by the control group levels. **(B)** Knockdown of *LINC01134* in HepG2 (left) and Huh-7 (right) HCC cells increased the expression of *CXCL2* and *CXCL3*, as detected by qRT-PCR. **(C)** Knockdown of *LINC01134* in HepG2 (left) and Huh-7 (right) HCC cells increased the migration of Jurkat T cells, as detected by the transwell assay. ns, *P* > 0.05; *P ≤ 0.05; **P ≤ 0.01; ***P ≤ 0.001.

To test the impact of *LINC01134* and *AC116025.2* on HCC, we measured their expression in two HCC cell lines using qRT-PCR and found that both *LINC01134* and *AC116025.2* were significantly upregulated in HepG2 and Huh-7 HCC cell lines compared to normal liver cell lines QSG-7701 and LO2 (**Figure 8A**). We also knocked down *LINC01134* expression in the two HCC cell lines and measured the expression of three cytokines whose expression correlated with *LINC01134* in HCC samples. *LINC01134* knockdown significantly increased the expression of *CXCL2* and *CXCL3* (**Figure 8B**). Consistent with the upregulation of *CXCL2* and *CXCL3* by *LINC01134* knockdown, conditioned medium from HCC cells with *LINC01134* knockdown significantly increased the migration of Jurkat T cells (**Figure 8C**). These findings support the role of *LINC01134* in HCC.

## Discussion

It is increasingly apparent that lncRNAs play crucial roles in the development and progression of cancers, including HCC, and TCE is a common mechanism for cancer cells to evade immune surveillance. In this study, we applied the recently developed TIDE program to available sequencing datasets of HCC to identify TCE-associated lncRNAs in HCC. Combing such lncRNAs with those differentially expressed in HCCs and subjecting them to additional statistical analyses, we developed an expression-based gene signature that predicts patient prognosis in HCC (**Figures 1**, **2**; **Table 1**). This signature consisted of 11 lncRNAs and was thus named 11 lncRNA prognostic signature (11LNCPS).

The 11LNCPS model appears to be robust. For example, the 11LNCPS score predicted patient OS in the training cohort of HCC and the validation and entire cohorts (**Figure 2D**). In addition, the discrimination power of the 11LNCPS was evident as the values of the area under ROC curves (AUC) for 1, 2, and 3 years were quite good in the training, validation, and entire cohorts of HCC (**Figure 2E**). Furthermore, the model’s C-index, which reflects predictive accuracy, was excellent, as indicated by values greater than 0.60 for 1, 2, and 3 years in each cohort (**Figure S1C**). The calibration curve demonstrated a good consistency for 1, 2, and 3 years in each cohort (**Figure S1D**).

The 11LNCPS model also appears to be more robust than two previously developed mRNA models, including the 8-gene model (34) and the 4-gene model (35). The 11LNCPS’s AUC values were equal or higher than those for the other two models in the validation cohort (**Figure 2F**), and so were the C-index values (**Figure S1E**).

Significantly, the 11LNCPS scores appear to predict the status of immune responses to HCC cells. Specifically, higher 11LNCPS scores were significantly associated with increased infiltrations of Th1, Th2, pro B, B, and basophils immune cells and decreased infiltrations of CD8+ Tcm, macrophages, M2 macrophage, aDCs, and cDCs immune cells in HCC (**Figure 3B** and **Figure S4**). Of them, decreased infiltration of CD8+ Tcm and increased infiltrations of Th1, Th2, and pro B cells were significantly correlated with worse patient OS in the same cohort of HCCs (**Figure 3A**). Additionally, higher 11LNCPS scores were associated with increased TCE (**Figure 3C**) and reduced T cell dysfunction (**Figure 3D**). Furthermore, HCCs with higher 11LNCPS scores significantly corresponded to malignancies that respond to PDL1 inhibition in immunotherapeutic studies, as analyzed by the SubMap program (**Figure 3E**). It is thus likely that HCCs with higher 11LNCPS scores respond better to immunotherapies than those with lower scores.

The 11LNCPS model was developed from differentially expressed and TCE-associated lncRNAs in HCC, so the impact of 11LNCPS scores on immune response and patient survival could be due to TCE to a greater extent. Many publications have reported the association of TCE with patient prognosis, tumor immune microenvironment, and treatment resistance (1, 11–15).

Immune cell infiltration to the tumor microenvironment determines the sensitivity of cancer cells to immunotherapy (50–53). In this regard, the 11LNCPS could predict the infiltration of cancer-related immune cells, as 11LNCPS scores were significantly associated with infiltration levels of 10 types of immune cells, and infiltration alterations in 7 of the 10 were associated with patient survival in HCC (**Figures 3A, B**). One major type is CD8+ T cells, whose infiltration was reduced in HCCs with higher 11LNCPS scores (**Figure 3B**). Reduced infiltration of CD8+ cells also occurred more frequently in HCCs with the upregulation of *LINC01134* or *AC116025.2* (**Figure 5C**). More importantly, reduced infiltration of CD8+ T cells, including naïve T and Tcm cells, negatively impacted patient survival in HCC (**Figure 3A**). Such an inverse correlation between the 11LNCPS score and the infiltration of CD8+ T cells further indicates the relevance of the 11LNCPS in HCC because CD8+ T cells play important roles in the killing of cancer cells. For example, CD8+ cytotoxic T lymphocytes (CTLs) kill cancer cells (54, 55); and CD8+ T cells, in total or in the form of naïve or memory cells, also play critically important roles in host defenses against tumor cells (38, 56). An inverse correlation between reduced CD8+ T cells and worse patient survival has been reported, although naïve T and Tcm cells were not distinguished in these studies (51, 54, 57–62).

Similar to CD8+ T cells, decreased infiltration of plasmacytoid dendritic cells (pDCs) was significantly associated with worse patient survival in HCC (**Figure 3A**), and a decrease in the infiltration of conventional DCs (cDCs) and activated DCs (aDCs) was more frequent in HCCs with higher 11LNCPS scores (**Figure 3B**). DCs play important roles in immune responses and tumor development. As antigen-presenting cells, pDCs function in adaptive immune responses to different antigens, including tumor antigens, and thus impact tumor development (63–65). Upon TRAIL-dependent mechanism and stimulation from other immune cells, activated pDCs indeed exert an anti-tumor function (57, 58, 66–68).

Opposing to the infiltrations of CD8+ cells and DCs, increased infiltration of CD4+ T helper cells, including Th1 and Th2 cells, and B cell progenitors (pro B) were significantly associated with worse patient survival and higher 11LNCPS scores in HCC (**Figures 3A, B**). Th1 and Th2 cells play important immunoregulatory roles in adaptive immunity, including the activation of B cells and cytotoxic T cells (69, 70). but their role in HCC development is not well understood (71). It is reported that a global Th1/Th2-like cytokine shift, i.e., an increase in Th2 cytokines but a decrease in Th1 cytokines, is associated with HCC metastasis (72), implicating Th1 and Th2 cells in HCC progression. We noticed that the association of Th1 cells with HCC prognosis is inconsistent between different studies (73). The role of pro B cells in HCC is not well understood either.

Immune cells’ infiltration into a tumor involves heterotypic signaling between tumor cells and immune cells. Such signaling is often mediated by chemokines, cytokines, and ICP ligands. Several such molecules could play roles in the 11LNCPS-associated modulation of the immune microenvironment in HCC. Taking advantage of the recently developed CellChat algorithm (26) and the availability of single-cell RNA sequencing (scRNA-seq) data of HCC (23), we were able to annotate CD8+ T cells. Subsequently, we identified the chemokines, cytokines, and ICP ligands that could mediate the recruitment of CD8+ T cells (**Figures S8A**, **B**). They included 22 chemokines and cytokines (**Figure 7A**, left) and 26 ICP ligands (**Figure 7A**, right). The expression of *LINC01134* was negatively correlated with that of *CXCL1*, *CXCL2*, *CXCL3*, *HLA-C*, and *HLA-E* but positively correlated with that of *LGALS9* (**Figure 7B**). Meanwhile, *AC116025.2* expression was positively correlated with *CXCL1*, *CXCL8*, *CXCL20*, and *TNFSF4* (**Figure 7C**). We could not annotate other types of 11LNCPS associated immune cells (e.g., Th1, Th2, etc.).

The 11 lncRNAs could impact multiple biological processes and signaling pathways in HCC. When HCCs with higher 11LNCPS scores were compared to those with lower scores, many processes and pathways were significantly enriched, particularly those of DNA replication, cell cycle, metabolism, signaling between cytokines and their receptors, and other ligand-receptor signaling pathways (**Figure 4**). Signaling pathways related to immune function and apoptosis were also significantly suppressed in HCCs with higher 11LNCPS scores, including the IL6-JAK-STAT3 signaling and IFNα response (**Figure 4C**).

Of the 11 lncRNAs in the 11LNCPS, *LINC01134* and *AC116025.2* appear more crucial than the others. For example, *LINC01134* and *AC116025.2* were among the 5 11LNCPS lncRNAs whose upregulation was significantly associated with worse patient OS in HCC (**Figure 5A**). In addition, the association of an upregulation with infiltration alteration was detected in more types of immune cells for *LINC01134* or *AC116025.2* than other lncRNAs (**Figures 5B, C**). Furthermore, HCCs with higher *LINC01134* or *AC116025.2* levels had significantly higher levels of TCE and lower scores of T cell dysfunction (**Figures 5D, E**). Increased TCE levels and reduced T cell dysfunction scores are associated with patient prognosis (37). LncRNA *LINC01134* has been well implicated in HCC, as it undergoes upregulation, promotes cell proliferation and invasion, suppresses apoptosis, and induces oxaliplatin resistance in HCC (74–77). Therefore, whereas *LINC01134* is more crucial in the 11LNCPS, there are hardly any published studies on *AC116025.2* in any types of cancers. The upregulation of both *LINC01134* and *AC116025.2* also occurs in HCC cell lines, as detected by qRT-PCR in HepG2 and Huh-7 HCC cells (**Figure 7D**).

*LINC01134* upregulation in HCC modulates multiple biological processes and signaling pathways (**Figure 6**). Of particular interest is that many of which are involved in immune functions, as *LINC01134* upregulation altered receptor-ligand activities, chemokine binding cellular component, chemokine signaling, cytokine and cytokine receptor, T and B cell receptor signaling, etc. (**Figure 6**). *LINC01134* upregulation also affects other cancer-related processes and pathways, including chromosome related molecular function, cell cycle and related pathways (E2F targets, G2M checkpoint, etc.), cancer-related pathways (PI3K-Akt, Rap1, MYC, etc.), cell death and related pathways (IFNγ response, IFNα response, etc.), IL6-JAK-STAT3 signaling, IL2- STAT5 signaling, epithelial-mesenchymal transition, UV response, TGFβ signaling, hypoxia, and P53 pathway (**Figure 6**). These findings further indicate that *LINC01134* impacts HCC *via* complicated signaling pathways, particularly those involved in immune functions. Consistent with these findings, *LINC01134* knockdown in HCC cell lines significantly increased the expression of chemokines *CXCL2* and *CXCL3* (**Figure 8B**), and conditioned medium from HCC cells with *LINC01134* knockdown increased the migration of T cells (**Figure 8C**).

Many *AC116025*-associated processes and pathways overlap with those of *LINC01134*, including receptor activity, cell cycle, metabolism, and cell death and related signaling pathways, E2F targets, G2M checkpoint, MYC, UV response, epithelial-mesenchymal transition, IFNγ response, IFNα response, UV response, epithelial-mesenchymal transition, bile acid metabolism, fatty acid metabolism, TGF-β signaling, etc. *AC116025.2* upregulation is less potent than *LINC01134* upregulation in its effects on immune-related processes and pathways. It did not significantly affect chemokine binding cellular component, chemokine signaling, cytokine and cytokine receptor, T and B cell receptor signaling, etc. (**Figure 6**).

Of note is that *AC116025.2* upregulation affects more metabolism-related pathways than *LNC001134* upregulation. In the KEGG pathway analysis, while 7 of the top 18 pathways affected by *AC116025.2* upregulation were metabolism-related, none of the top 14 affected by *LNC001134* were (**Figures 6B, D**), even though they both affected bile acid metabolism and fatty acid metabolism in the GSEA enrichment assay (**Figures 6C, F**). Tumor cell metabolism reprograms immune cell infiltration (78, 79), so the association of *AC116025.2* with alterations in multiple metabolic pathways could suggest how *AC116025.2* might modulate T cell exclusion.

In summary, after identifying differentially expressed and TCE-associated lncRNAs in HCC, we developed and validated a robust lncRNA-based gene signature named 11LNCPS for 11-lncRNA prognosis signature. The 11LNCPS predicts not only prognosis but also immune cells’ responses to tumor cells, including decreased infiltrations of CD8+ T cells, macrophages, and DCs, as well as increased infiltrations of Th1, Th2, pro B cells. Of the 11 lncRNAs in the 11LNCPS, *LINC01134* and *AC116025.2* appear more crucial than the others. Expression alterations in the 11LNCPS lncRNAs, particularly the upregulation of *LINC01134* and *AC116025.2*, modulate multiple signaling pathways, including immune responses and cell metabolism. The 11LNCPS could help predict immune responses in HCC and provide candidate therapeutic targets for the treatment of HCC.

## Data Availability Statement

Publicly available datasets were analyzed in this study. This data can be found in TCGA (https://www.cancer.gov/about-nci/organization/ccg/research/structural-genomics/tcga/) and GEO (https://www.ncbi.nlm.nih.gov/geo/) database.

## Author Contributions

JD, XL, ZZ, and ML contributed to conception and design of the study. XL and XF curated the data. XL performed the statistical analysis. XL wrote the first draft of the manuscript. JD, JA, ZZ, GC, and SW edited and wrote sections of the manuscript. JD supervised the study. All authors contributed to manuscript revision, read, and approved the submitted version.

## Funding

This study is supported in part by grant JCYJ20200109141229255 from the Science, Technology and Innovation Commission of Shenzhen Municipality.

## Conflict of Interest

The authors declare that the research was conducted in the absence of any commercial or financial relationships that could be construed as a potential conflict of interest.

## Publisher’s Note

All claims expressed in this article are solely those of the authors and do not necessarily represent those of their affiliated organizations, or those of the publisher, the editors and the reviewers. Any product that may be evaluated in this article, or claim that may be made by its manufacturer, is not guaranteed or endorsed by the publisher.
