# Supplementary material for: Establishment of a lncRNA-Based Prognostic Gene Signature Associated With Altered Immune Responses in HCC
Source: Front Immunol. 2022 Apr 28;13:880288. doi: 10.3389/fimmu.2022.880288 (PMC9097819; doi:10.3389/fimmu.2022.880288)
Supplement: Supplementary file 1 [file DataSheet_1.pdf]

# *Supplementary Material*

## 1 Supplementary Figures and Tables

### 1.1 Supplementary Figures

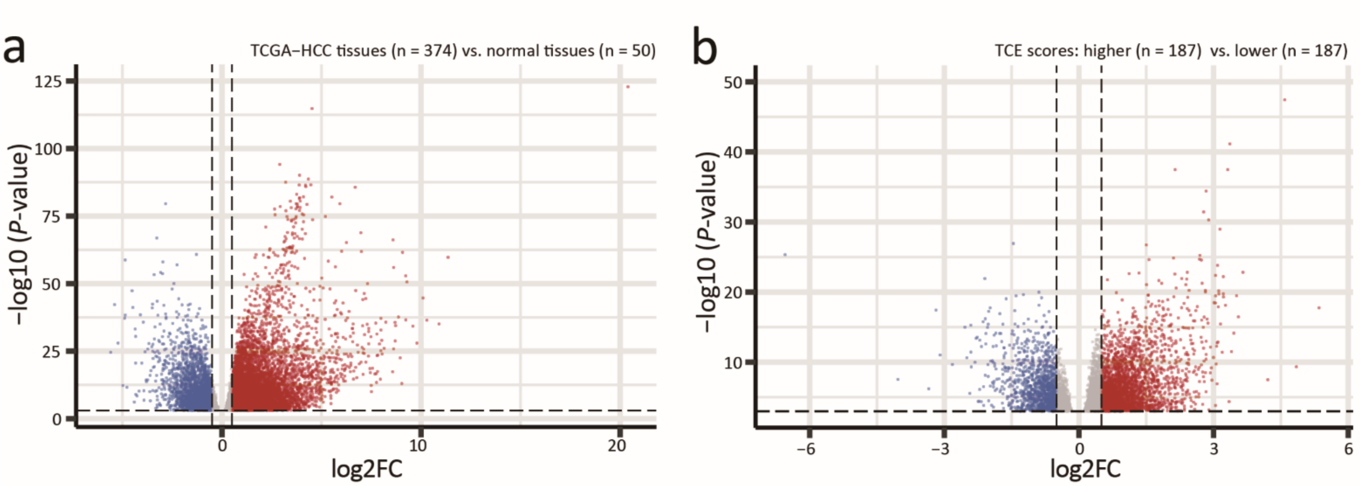


**Figure S1.** Genes differentially expressed between HCC and normal liver tissues (**a**) and between HCCs with higher and lower T cell exclusion levels (**b**), as shown in volcano plots. The X-axis represents log2FC and the Y-axis represents -log10 (adjusted *P*-value).

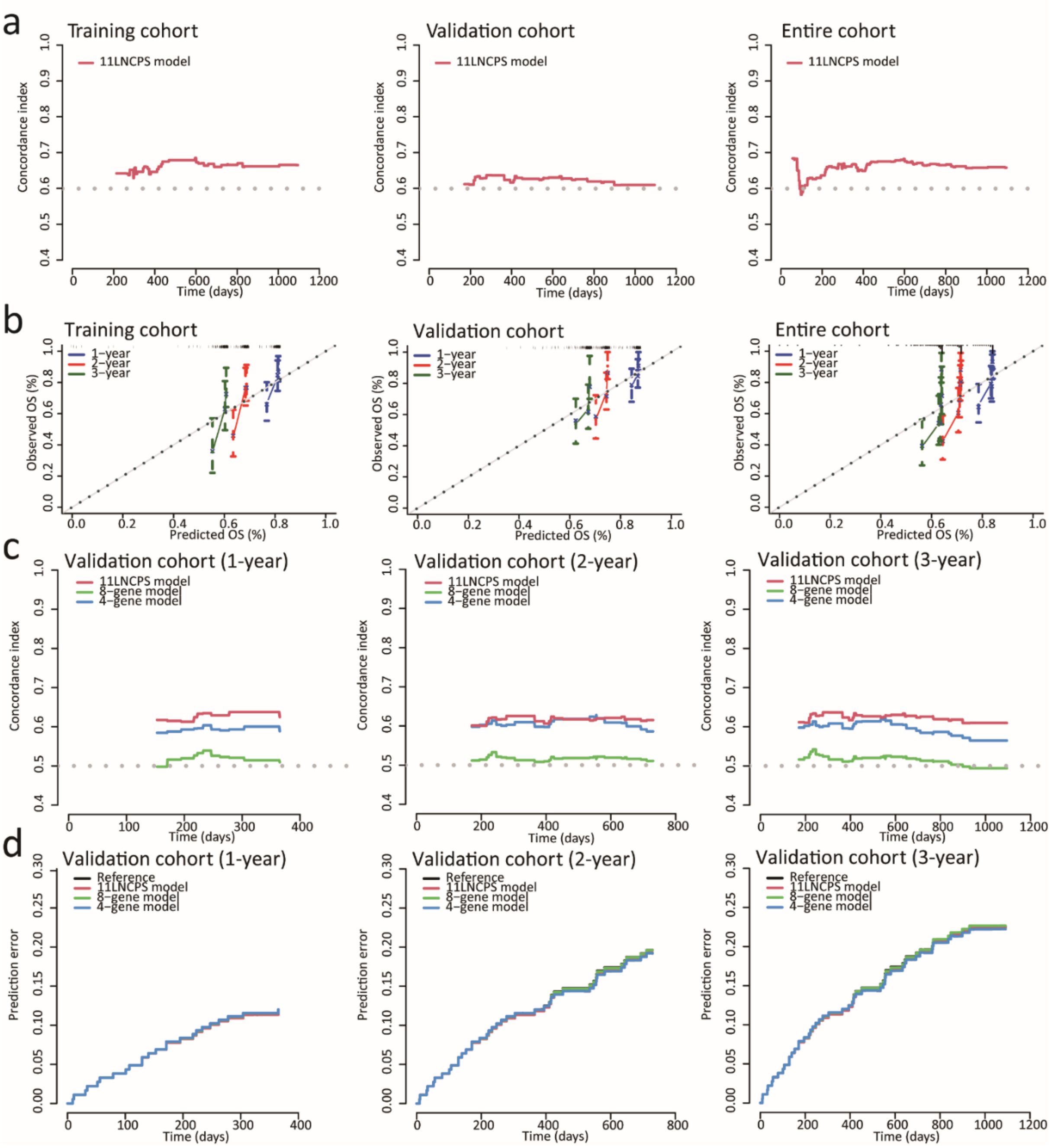


**Figure S2. Validation of the 11LNCPS model by different approaches.** (**a**) Prediction of the concordance index (C-index) curves of 3-year overall survival (OS) by the 11LNCPS model in the training cohort (left), validation cohort (middle), or entire cohort (right). (**b**) Calibration curves of the 11LNCPS model for predicting the OS probability at 1, 2, and 3 years in the training cohort (left), validation cohort (center), and entire cohort (right). (**c**) Comparison of C-index curves of 1, 2, and 3-year OS predicted by the 11LNCPS model and two previously published models in the validation cohort. (**d**) Comparison of prediction error curves of 1, 2, and 3-year OS predicted by the 1LNCPS model and two previously published models in the validation cohort.

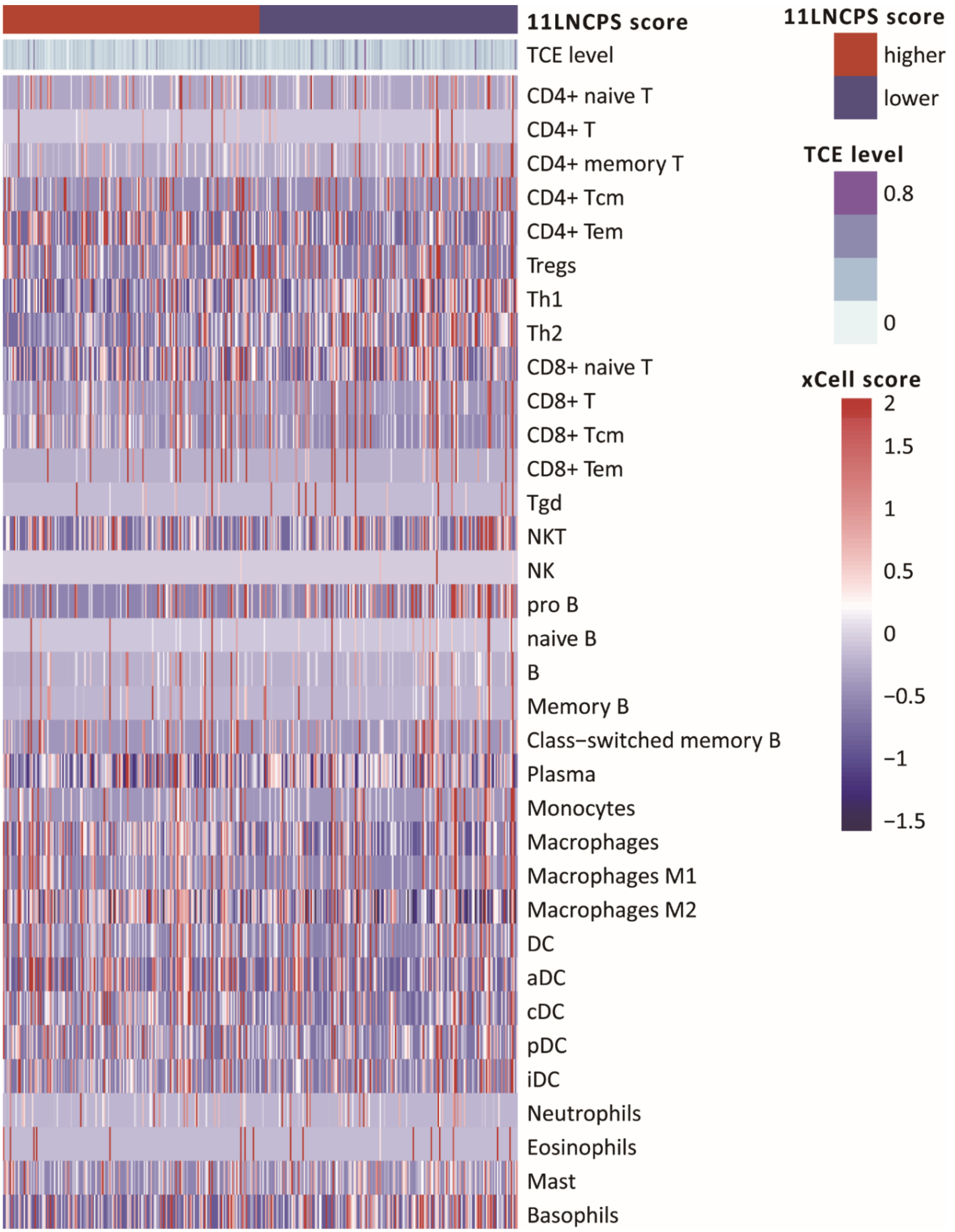


**Figure S3. Comparison of infiltration status of 34 types of immune cells between HCCs with higher and lower 11LNCPS scores.** The abundance of each immune cell type was estimated by the xCell assay and is shown in xCell scores and visualized in a heatmap. TCE scores are also shown, and their levels are indicated by gradient color bars at right.

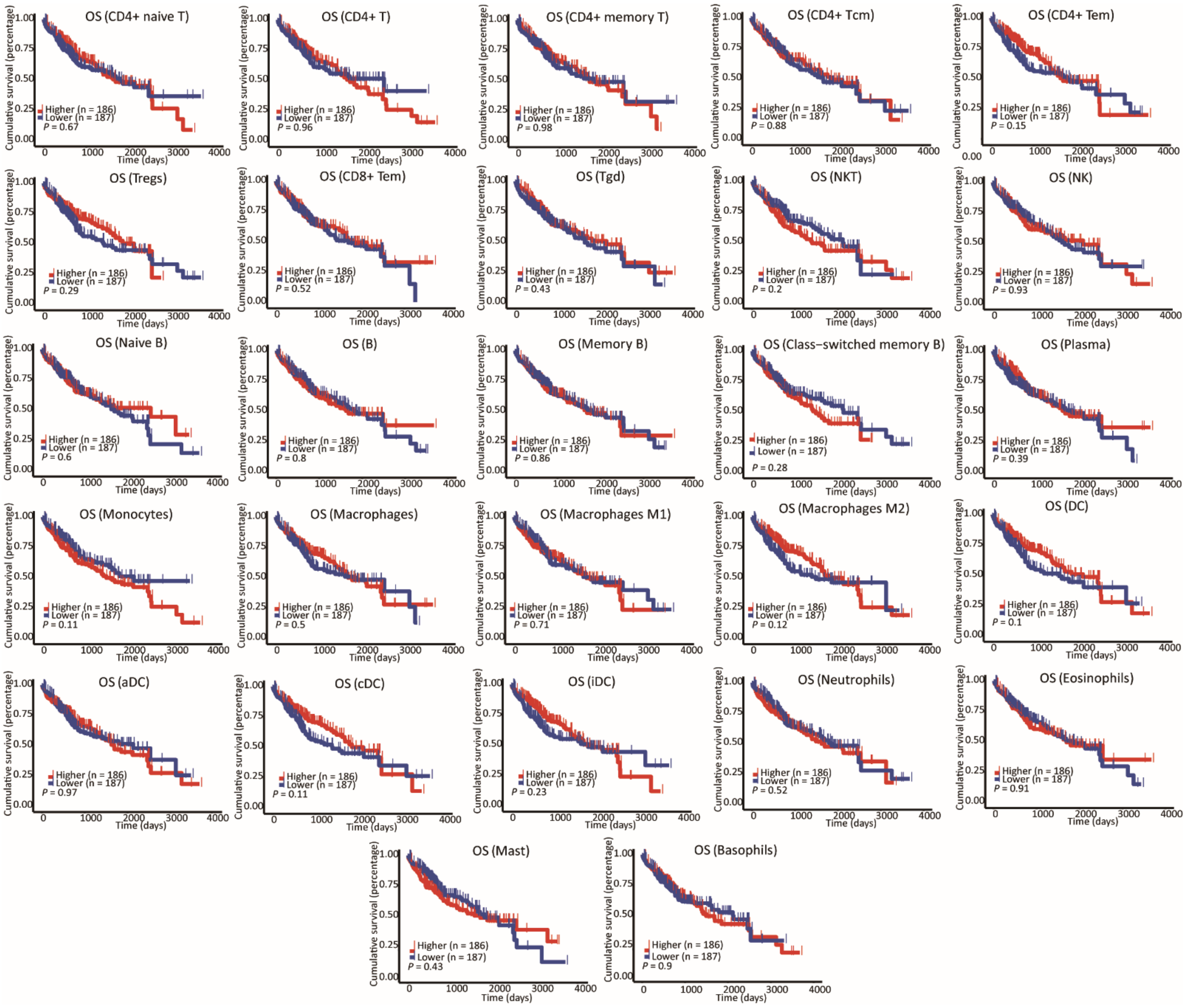


**Figure S4. Types of immune cells whose infiltration did not correlate with patient overall survival (OS) in HCC.** The Kaplan-Meier analysis was used to determine the correlation.

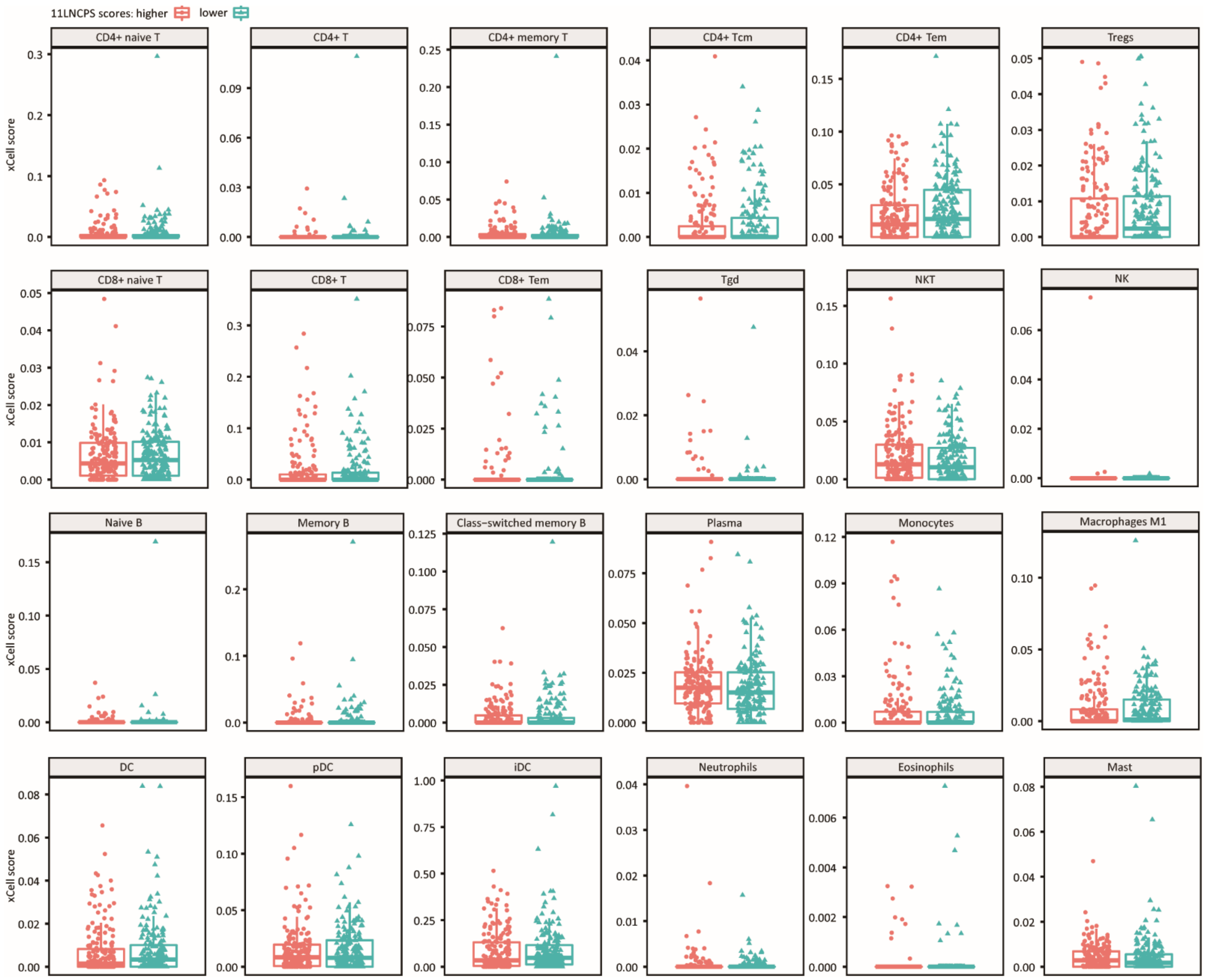


**Figure S5. Types of immune cells whose infiltration level did not differ between HCCs with higher 11LNCPS scores and those with lower scores.** The Wilcoxon test was used for the analysis.

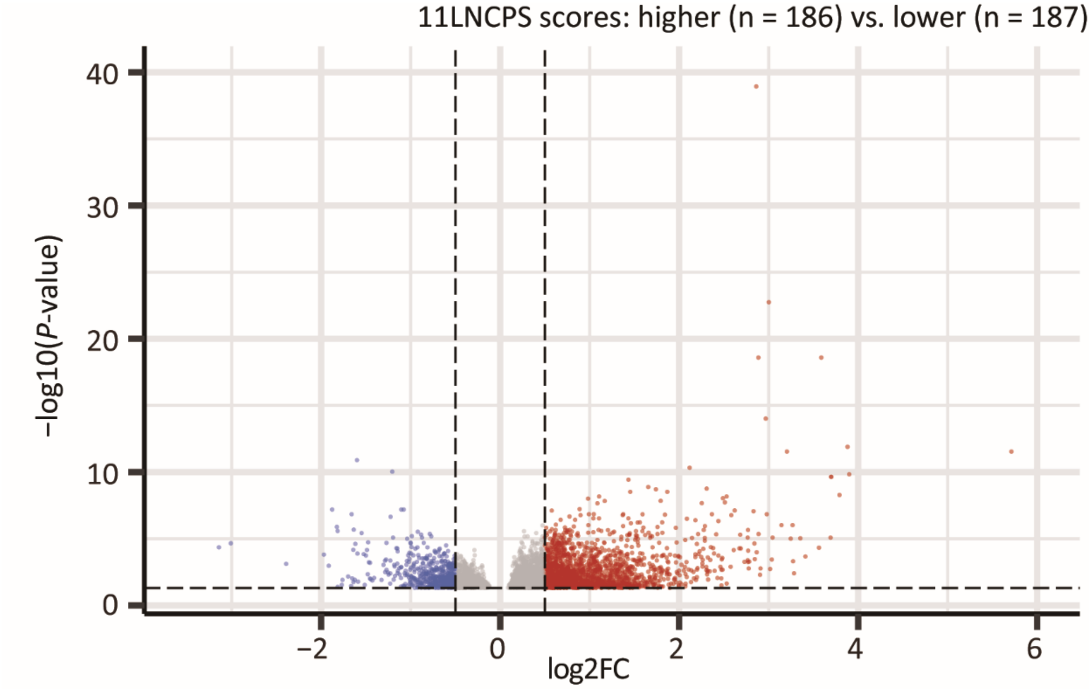


**Figure S6. Volcano plot of differentially expressed genes between HCCs with higher and lower 11LNCPS scores.** X-axis represents log2FC and the Y-axis represents -log10 (adjusted *P*-value).

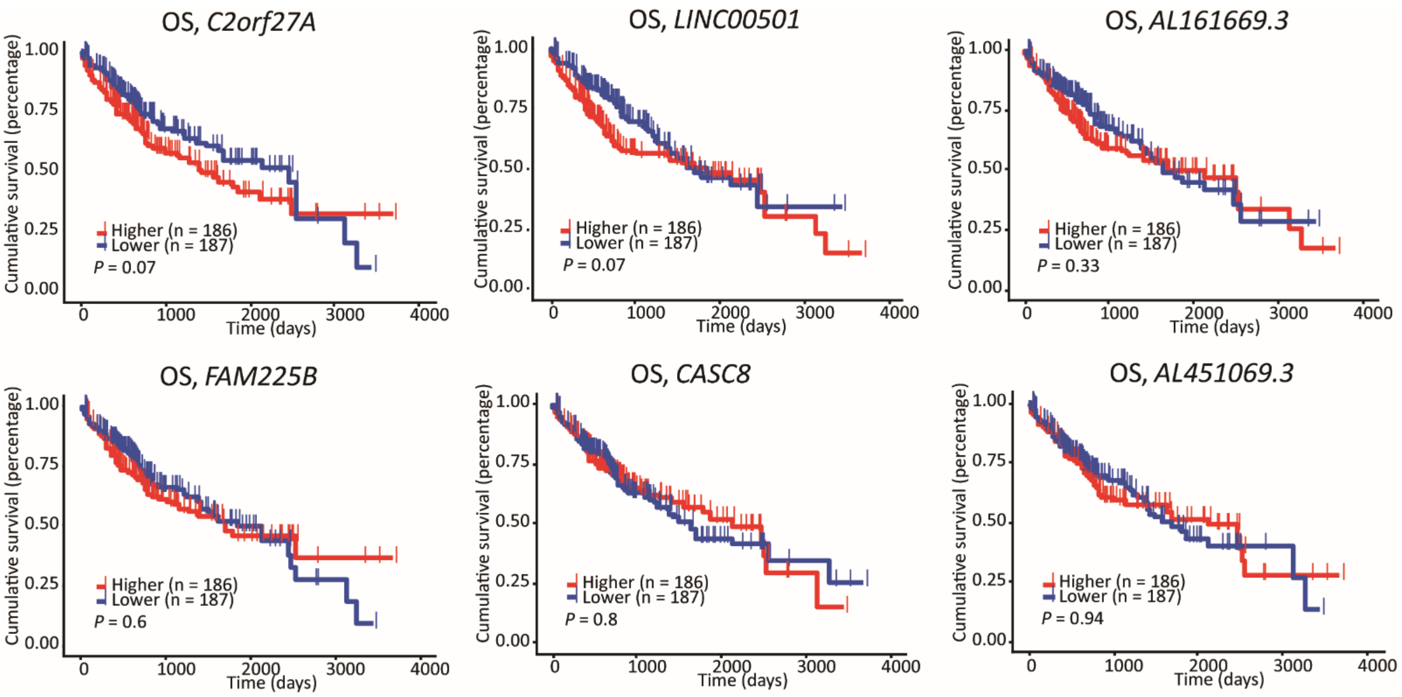


**Figure S7. LncRNAs of the 11LNCPS that do not have a significant correlation with overall survival (OS) in HCC.** Kaplan-Meier survival analysis was used for the determination.

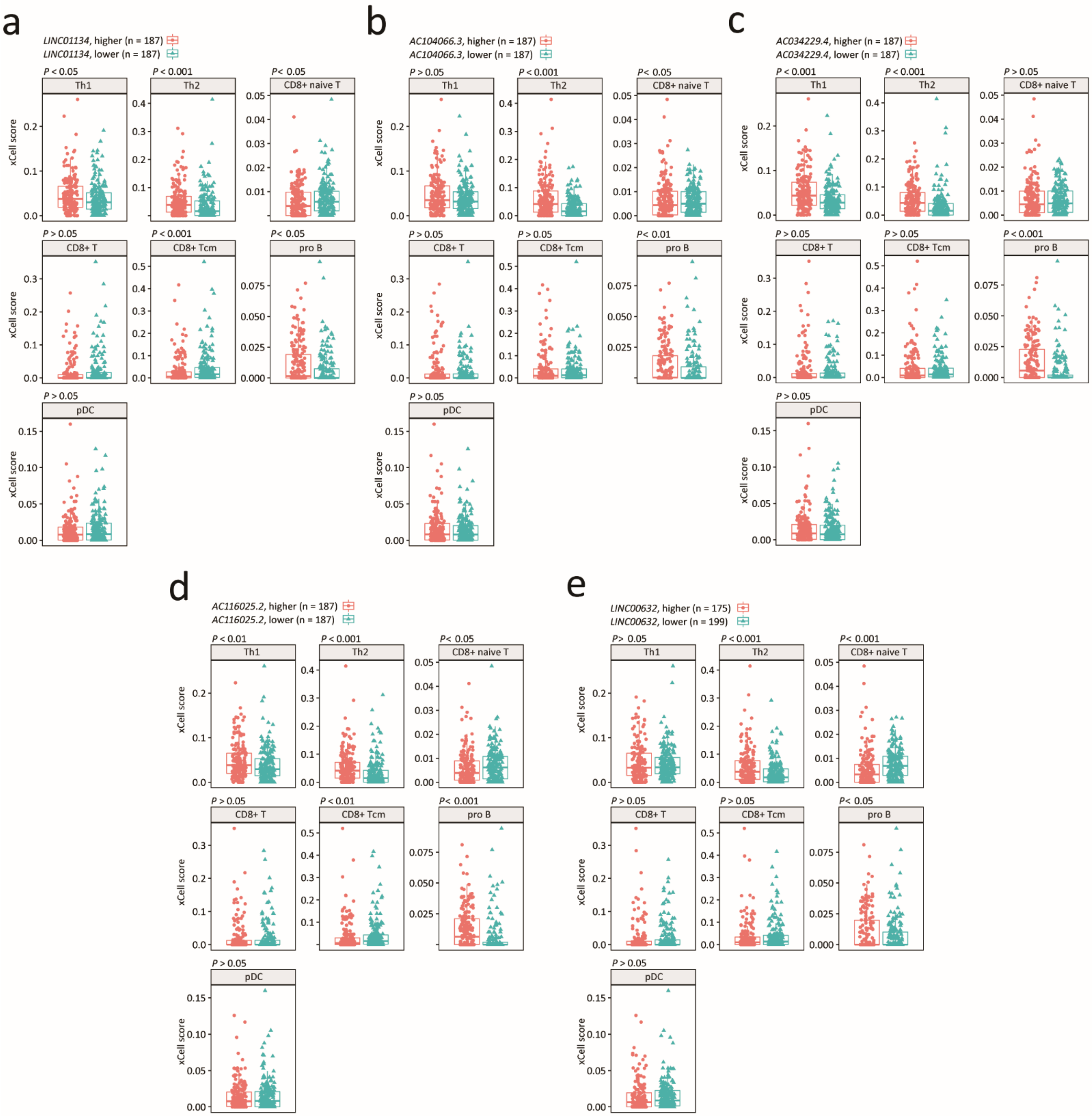


**Figure S8. Comparison of infiltration levels of prognosis-associated immune cell types between HCCs with higher and lower expression levels of a prognosis-associated 11LNCPS lncRNA.** The Wilcoxon test was used to compare, and the results are shown in box plots. The prognosis-associated 11LNCPS lncRNAs include *LINC01134* (**a**), *AC104066.3* (**b**), *AC034229.4* (**c**), *AC116025.2* (**d**), and *LINC00632* (**e**). The infiltration levels are indicated by xCell scores. HCCs were divided into higher and lower groups for each lncRNA using its median expression level.

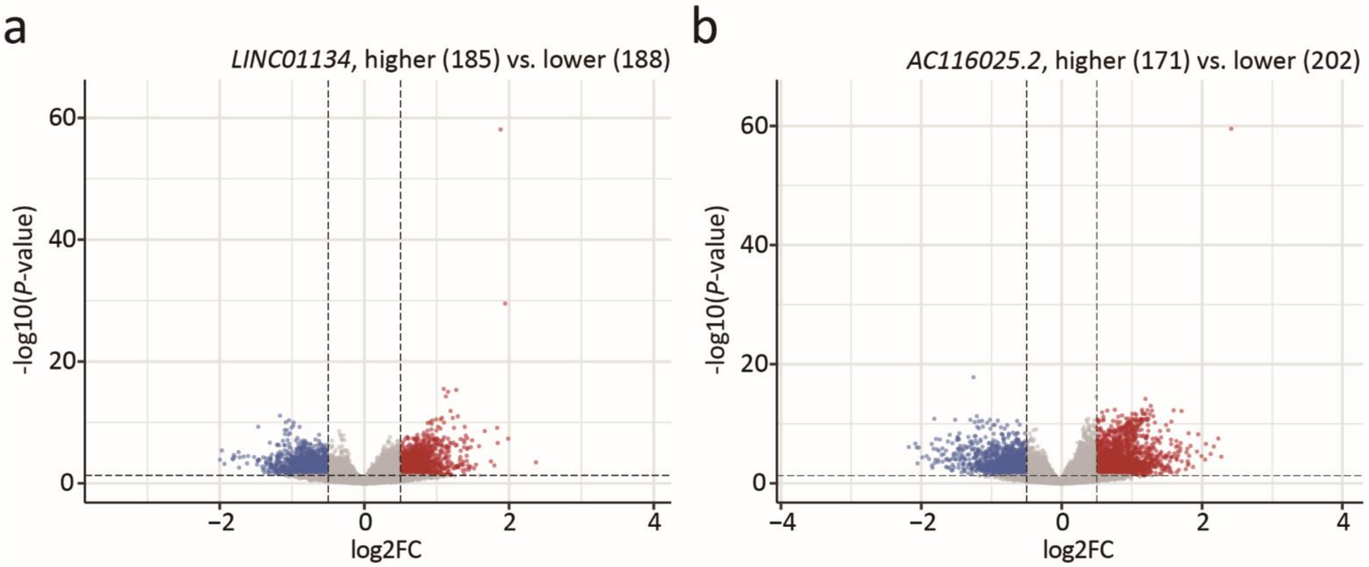


**Figure S9. Volcano plots of differentially expressed genes between HCCs with higher and lower *LINC01134* or *AC116025.2*.** The X-axis represents log2FC and the Y-axis represents -log10 (adjusted *P*-value).

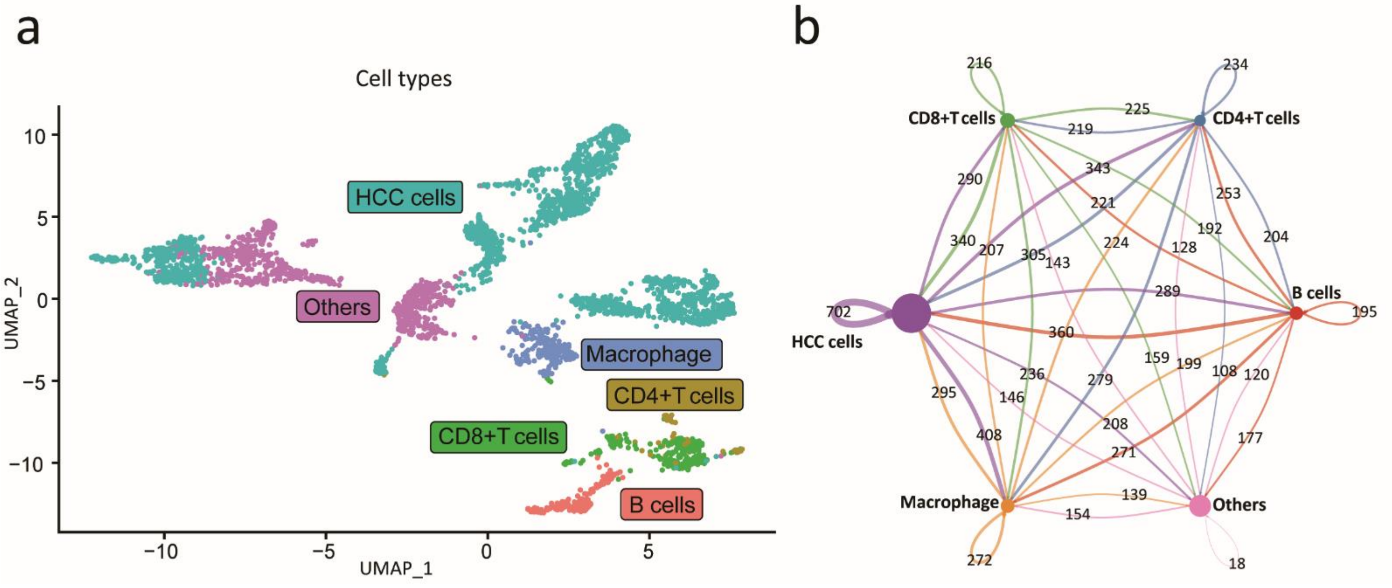


**Figure S10. Heterotypic signaling crosstalk between HCC cells and immune cells estimated by single-cell RNA sequencing analysis.** (**a**) Identification of cancer and immune cell types by single-cell sequencing analysis of HCCs, as shown in the UMAP plot. (**b**) Cell-cell communication network among cancer and immune cell types identified in panel A, as established by the CellChat analysis. In the circle plot, each line indicates crosstalk between two cell types based on a ligand-receptor interaction, and the number on a line indicates the number of ligand-receptor pairs.

## 1.2 Supplementary table

**Table S1. LncRNAs that are differentially expressed and TCE-associated in HCCs (n = 336)**. Shown for each lncRNA include gene symbol, ENSEMBL ID, chromosomal location (Chr.), sequence position in a chromosome (start and end), hazard ratio (HR) for survival and its 95% confidence interval from low to high (HR.95L-HR.95H) expression fold change in log2 and its adjusted *P* value, and TCE fold change in log2 and its adjusted *P* value. Asterisks indicate the 54 lncRNAs whose expression alterations were significantly associated with overall survival in the training cohort.

| SYMBOL | ENSEMBL ID | Chr. | Start | End | HR | HR.95L-HR.95H | *P* value | Expression fold change | *P* value | TCE fold change | *P* value |
|---|---|---|---|---|---|---|---|---|---|---|---|
| *LINC02593* | ENSG00000223764 | 1 | 916865 | 921016 | 0.316694 | 0.055174436-1.817784769 | 0.1971681 | -1.6356429 | 1.17E-06 | 0.94219 | 0.000259 |
| *AL645608.7* | ENSG00000272512 | 1 | 995966 | 998051 | 0.992545 | 0.82096842-1.199979372 | 0.9384012 | 0.90744971 | 0.000636 | 1.236128 | 2.78E-12 |
| *LINC01134** | ENSG00000236423 | 1 | 3900352 | 3917225 | 2.957099 | 1.490261982-5.8677175 | 0.0019284 | 1.25384639 | 3.36E-12 | 0.556164 | 1.83E-05 |
| *LINC02766* | ENSG00000229484 | 1 | 12527724 | 12528420 | 0.903351 | 0.699379724-1.166808772 | 0.4363015 | -0.8638034 | 8.00E-05 | -0.76884 | 1.48E-06 |
| *AL121992.3* | ENSG00000272510 | 1 | 15565611 | 15565956 | 1.0809 | 0.998277796-1.170360857 | 0.0551772 | -0.771452 | 0.000131 | -0.80305 | 1.58E-08 |
| *SNHG3** | ENSG00000242125 | 1 | 28505980 | 28510892 | 1.080353 | 1.024245347-1.139533346 | 0.0045062 | 1.67351936 | 2.46E-21 | 0.84009 | 1.54E-11 |
| *AL357079.1** | ENSG00000237950 | 1 | 43937918 | 43946551 | 1.87064 | 1.143125436-3.061165206 | 0.0126924 | 1.42599502 | 5.45E-12 | 0.623872 | 2.80E-07 |
| *AL592166.1* | ENSG00000225721 | 1 | 44759037 | 44775810 | 1.975124 | 0.649544515-6.005924198 | 0.2303229 | 1.14412896 | 6.64E-08 | 0.542804 | 1.38E-05 |
| *LINC01389* | ENSG00000225762 | 1 | 47380928 | 47408477 | 1.06031 | 0.849051567-1.32413227 | 0.6054643 | 1.8929204 | 1.75E-15 | -0.75632 | 2.43E-06 |
| *LINC01767* | ENSG00000223956 | 1 | 56414935 | 56417137 | 0.998529 | 0.983602542-1.013682678 | 0.8481213 | -1.5757693 | 5.52E-11 | -1.14866 | 1.38E-11 |
| *AL161740.1* | ENSG00000236341 | 1 | 56963886 | 56996757 | 0.880214 | 0.541592462-1.430552138 | 0.6066069 | -1.641166 | 5.81E-15 | -0.64658 | 8.15E-05 |
| *LINC01702* | ENSG00000235200 | 1 | 67522299 | 67532612 | 0.989267 | 0.972275419-1.006555031 | 0.2221564 | -1.4439763 | 2.39E-05 | -1.08018 | 1.76E-05 |
| *AL583808.1* | ENSG00000225087 | 1 | 72636547 | 72899240 | 1.271942 | 0.84603444-1.912257995 | 0.2475692 | 3.33919665 | 2.49E-05 | 1.698588 | 0.000954 |
| *LINC00622* | ENSG00000260941 | 1 | 119597702 | 119599271 | 1.36356 | 0.983028379-1.891396773 | 0.0632493 | 1.54156786 | 8.45E-12 | 0.815519 | 2.80E-08 |
| *AL592494.1* | ENSG00000228826 | 1 | 121494329 | 121511588 | 1.029473 | 0.973937159-1.088175256 | 0.304606 | -0.8369263 | 0.000172 | -0.90382 | 9.96E-09 |

| | | | | | | | | | | | |
|---|---|---|---|---|---|---|---|---|---|---|---|
| *LINC02802** | ENSG00000232527 | 1 | 144227029 | 144250326 | 1.785999 | 1.278897676-2.494173334 | 0.000665 | 2.43502519 | 5.99E-14 | 0.869513 | 2.55E-05 |
| *AL356356.1** | ENSG00000237781 | 1 | 150548562 | 150557724 | 1.180382 | 1.010292802-1.379107934 | 0.036712 | 1.59202404 | 1.91E-08 | 0.579524 | 0.000252 |
| *AL355388.1* | ENSG00000224276 | 1 | 155991390 | 156001787 | 3.581166 | 0.912334442-14.05707034 | 0.0674806 | 2.54655706 | 1.26E-16 | 0.738265 | 5.63E-05 |
| *SNHG28* | ENSG00000188004 | 1 | 159834495 | 159855237 | 1.005489 | 0.297047169-3.403528222 | 0.9929794 | 1.26717093 | 9.22E-07 | 0.739007 | 2.90E-05 |
| *AL590560.3* | ENSG00000272668 | 1 | 159854870 | 159867685 | 1.168163 | 0.736262128-1.853422269 | 0.5092748 | 1.03571529 | 0.000851 | 0.935553 | 4.05E-06 |
| *LINC00862* | ENSG00000203721 | 1 | 200253419 | 200400705 | 1.167023 | 0.728979746-1.868286277 | 0.5200106 | 1.64561336 | 3.03E-11 | 0.593921 | 0.000803 |
| *AL358473.1* | ENSG00000229191 | 1 | 201023949 | 201028792 | 242.6289 | 0.196410052-299723.9922 | 0.1305642 | 2.50381626 | 2.15E-08 | 1.200571 | 1.78E-06 |
| *AL391832.1* | ENSG00000237520 | 1 | 234957231 | 234959989 | 0.937322 | 0.783115257-1.121894449 | 0.4803135 | 2.41116125 | 5.80E-12 | -0.93983 | 5.60E-05 |
| *LINC01348* | ENSG00000280587 | 1 | 235065479 | 235074220 | 1.015183 | 0.969429362-1.063096491 | 0.5218884 | -1.1609267 | 5.34E-05 | -1.33613 | 4.06E-11 |
| *AC114810.1* | ENSG00000271868 | 2 | 3496956 | 3497428 | 0.947382 | 0.814223435-1.102317659 | 0.4842816 | -0.8760405 | 4.70E-07 | -0.59407 | 2.73E-06 |
| *U91324.1* | ENSG00000229740 | 2 | 8139335 | 8144950 | 1.023842 | 0.940355436-1.114739941 | 0.5871841 | -1.2856383 | 1.91E-08 | -0.79493 | 2.75E-06 |
| *AC010969.1* | ENSG00000188525 | 2 | 10001757 | 10006030 | 1.088796 | 0.859907482-1.378608816 | 0.4798705 | -1.8967632 | 9.03E-06 | -1.09101 | 0.00038 |
| *AC115619.1* | ENSG00000261012 | 2 | 20999313 | 21000917 | 1.000219 | 0.986575642-1.014051801 | 0.9750283 | -1.0568876 | 3.21E-07 | -1.1284 | 2.70E-15 |
| *AC018467.1* | ENSG00000232451 | 2 | 23018125 | 23199056 | 0.962163 | 0.795036963-1.16442112 | 0.6919373 | -1.0434734 | 8.33E-05 | -0.82785 | 2.36E-05 |
| *AC104695.2* | ENSG00000229951 | 2 | 28384409 | 28394672 | 0.370234 | 0.028569086-4.797964137 | 0.4471422 | 1.69252815 | 3.21E-06 | 0.79752 | 0.000812 |
| *AC073283.2* | ENSG00000226087 | 2 | 47225781 | 47240886 | 1.053952 | 0.878778636-1.264042772 | 0.5709897 | 1.55051577 | 0.00057 | -1.43863 | 3.81E-06 |
| *AC007099.1* | ENSG00000231172 | 2 | 75524068 | 75542706 | 1.012033 | 0.987246813-1.037441683 | 0.3444325 | 3.14773303 | 2.65E-10 | 1.843188 | 3.61E-09 |
| *AC092683.1** | ENSG00000230606 | 2 | 97416165 | 97433527 | 70.74853 | 1.311361912-3816.913004 | 0.036333 | 1.86319827 | 2.94E-10 | 0.755957 | 8.33E-06 |
| *C2orf27A** | ENSG00000287151 | 2 | 131647990 | 131767404 | 1.713252 | 1.301972612-2.25444941 | 0.000121 | 1.16166071 | 9.60E-08 | 1.056246 | 6.00E-13 |

| | | | | | | | | | | | |
|---|---|---|---|---|---|---|---|---|---|---|---|
| *LINC01818* | ENSG00000230645 | 2 | 150169474 | 150356460 | 0.827751 | 0.305588794-2.242135036 | 0.7100196 | -2.6000263 | 8.27E-12 | -1.0926 | 0.000713 |
| *LINC02612* | ENSG00000224048 | 2 | 150612381 | 150635818 | 3.100306 | 0.00148868-6456.654095 | 0.7716456 | -1.3415325 | 0.000214 | -1.32966 | 2.14E-06 |
| *AC007277.1* | ENSG00000213981 | 2 | 170640374 | 170695374 | 0.544634 | 0.201945599-1.46884407 | 0.2299773 | 3.67814814 | 8.68E-16 | 1.371244 | 1.97E-06 |
| *LINC01116* | ENSG00000163364 | 2 | 176629572 | 176637931 | 1.287684 | 0.992598625-1.670494074 | 0.0569074 | 3.10351045 | 3.25E-22 | 1.020994 | 1.77E-06 |
| *AC005540.1* | ENSG00000235852 | 2 | 190880797 | 190882059 | 1.041239 | 0.010572109-102.5507637 | 0.9862324 | 1.25953823 | 0.000765 | 0.727555 | 0.000689 |
| *AC097717.1* | ENSG00000232732 | 2 | 199867396 | 199911159 | 1675.204 | 0.003602542-778979770.4 | 0.2648625 | 1.5260865 | 3.11E-05 | 0.678658 | 0.000852 |
| *AC098820.2* | ENSG00000235042 | 2 | 216385288 | 216412696 | 1.773667 | 0.934495067-3.366409528 | 0.0796458 | 1.47253112 | 3.66E-15 | 0.512669 | 6.03E-05 |
| *AC016717.2* | ENSG00000273301 | 2 | 225698514 | 225703654 | 0.786779 | 0.415291176-1.490572718 | 0.4619844 | 4.96428288 | 1.88E-10 | 2.426174 | 1.01E-06 |
| *AC009407.1** | ENSG00000261829 | 2 | 231052040 | 231052637 | 1.027885 | 1.005407635-1.050865304 | 0.0147676 | 0.81960217 | 5.17E-05 | -0.55738 | 0.000183 |
| *AC104809.1* | ENSG00000223991 | 2 | 240981515 | 240986072 | 1.000135 | 0.942405771-1.061400655 | 0.9964482 | -2.3499808 | 5.98E-06 | -1.97353 | 2.48E-07 |
| *AC006252.1** | ENSG00000243224 | 3 | 52239258 | 52241097 | 4.890076 | 1.117829024-21.39221464 | 0.0350402 | 2.02873971 | 2.22E-16 | 0.539802 | 6.36E-05 |
| *AC119424.1* | ENSG00000243384 | 3 | 58572744 | 58574319 | 1.107275 | 0.783949436-1.563949594 | 0.5630036 | -1.7067544 | 4.64E-09 | -0.77731 | 0.000498 |
| *AC097634.1* | ENSG00000270562 | 3 | 71584943 | 71587409 | 4.016383 | 0.093514806-172.5003204 | 0.4686012 | 1.77009717 | 1.56E-09 | 0.653731 | 0.000107 |
| *AC063938.1* | ENSG00000241754 | 3 | 102621061 | 102673986 | 1.148793 | 0.50679176-2.604076912 | 0.7397306 | -1.661155 | 7.41E-10 | -0.84039 | 9.56E-05 |
| *LINC00488* | ENSG00000214381 | 3 | 109178143 | 109216965 | 0.707382 | 0.374322063-1.336786484 | 0.2863877 | 3.43714611 | 2.89E-10 | 1.976464 | 1.19E-07 |
| *LINC00880* | ENSG00000243629 | 3 | 157081667 | 157128595 | 0.763723 | 0.411559751-1.417223338 | 0.3928147 | 1.90812631 | 1.07E-08 | -0.90082 | 0.000142 |
| *AC080013.3* | ENSG00000271778 | 3 | 158782547 | 158783124 | 3.165136 | 0.934137312-10.72442208 | 0.0642361 | 1.61565018 | 7.77E-09 | 0.532467 | 0.000749 |
| *LINC00501** | ENSG00000203645 | 3 | 177294442 | 177323418 | 5.639399 | 2.471580449-12.86740252 | 3.96E-05 | 3.1162168 | 3.46E-09 | 1.392693 | 1.45E-05 |
| *LINC02028* | ENSG00000230102 | 3 | 194005259 | 194071025 | 1.345676 | 0.623256752-2.905452582 | 0.4496351 | -0.7911291 | 0.000292 | -0.931 | 1.30E-09 |
| *LINC00887* | ENSG00000214145 | 3 | 194296191 | 194322871 | 1.403048 | 0.945561831-2.081876825 | 0.0925787 | 1.51439067 | 3.53E-06 | 1.15212 | 1.74E-07 |

| | | | | | | | | | | | |
|---|---|---|---|---|---|---|---|---|---|---|---|
| *AC024560.3* | ENSG00000273308 | 3 | 197645669 | 197646017 | 1.521166 | 0.805179376-2.87382732 | 0.1962309 | 1.403065 | 1.25E-06 | 0.717006 | 1.23E-06 |
| *AC092535.5* | ENSG00000273179 | 4 | 1167778 | 1168174 | 1.012822 | 0.990708629-1.035428583 | 0.2579865 | 1.91995596 | 9.94E-21 | 0.513973 | 0.000773 |
| *LINC02600* | ENSG00000250986 | 4 | 3758748 | 3763390 | 0.92113 | 0.503490525-1.685194821 | 0.7897974 | 2.59798189 | 6.96E-12 | 1.371764 | 2.14E-07 |
| *AC005699.1* | ENSG00000249631 | 4 | 11625714 | 11823906 | 4.203809 | 0.205167031-86.13473892 | 0.3513496 | 1.80297051 | 0.000404 | 1.239791 | 9.39E-05 |
| *AC104066.3** | ENSG00000260120 | 4 | 52680609 | 52692999 | 7.467815 | 1.969993365-28.30885859 | 0.0031043 | 1.30910446 | 2.76E-05 | 1.028498 | 1.70E-08 |
| *DANCR** | ENSG00000226950 | 4 | 52712404 | 52720351 | 1.020597 | 1.00247452-1.03904799 | 0.0257259 | 0.73015913 | 6.80E-05 | 0.646968 | 8.21E-07 |
| *AC107396.1* | ENSG00000251049 | 4 | 57595940 | 57605872 | 0.666353 | 0.164421618-2.700536778 | 0.5696626 | -4.3289586 | 1.05E-09 | -3.34606 | 6.00E-07 |
| *LINC01094* | ENSG00000251442 | 4 | 78638780 | 78683185 | 1.529855 | 0.859363327-2.72347792 | 0.1484873 | 0.8270101 | 0.000736 | 0.584433 | 0.00079 |
| *LINC01088* | ENSG00000249307 | 4 | 78939485 | 79309673 | 2.878004 | 0.235863256-35.11741074 | 0.4075466 | 1.62248655 | 5.87E-05 | 1.039377 | 3.30E-05 |
| *AC114811.2* | ENSG00000260641 | 4 | 98658904 | 98664550 | 0.018146 | 5.06E-05-6.50198711 | 0.18152 | 2.72815237 | 4.28E-09 | 1.233649 | 2.16E-05 |
| *AC083902.1* | ENSG00000248676 | 4 | 99594799 | 99625913 | 1.072107 | 0.797857416-1.440625435 | 0.6441641 | -0.668058 | 0.000719 | -0.5497 | 0.000199 |
| *LINC02428* | ENSG00000248740 | 4 | 103255822 | 103454667 | 1.029471 | 0.998351991-1.061560915 | 0.0636467 | -1.9715658 | 2.67E-09 | -1.48233 | 6.37E-10 |
| *AC096564.1* | ENSG00000245293 | 4 | 107863473 | 107989679 | 1.109764 | 0.025090088-49.08618803 | 0.957041 | -1.3029661 | 4.68E-08 | -1.35868 | 8.10E-16 |
| *AC078850.1** | ENSG00000248187 | 4 | 128567972 | 128570531 | 11.81261 | 1.628360623-85.69216461 | 0.0145974 | 2.39425337 | 2.80E-08 | 1.228353 | 2.84E-07 |
| *LINC02362* | ENSG00000249096 | 4 | 184365180 | 184382416 | 0.938059 | 0.852377769-1.032352834 | 0.1907288 | -1.2195095 | 1.21E-05 | -1.18696 | 1.82E-09 |
| *LINC01093* | ENSG00000249173 | 4 | 184892858 | 184899553 | 1.010119 | 0.987283458-1.033481892 | 0.3881566 | -3.3965733 | 6.15E-20 | -1.91164 | 5.67E-13 |
| *LINC01018* | ENSG00000250056 | 5 | 6582136 | 6600288 | 0.9965 | 0.98289692-1.010291495 | 0.617112 | -1.4802897 | 7.75E-05 | -1.17819 | 2.24E-05 |
| *AC034229.4** | ENSG00000272417 | 5 | 10203600 | 10204040 | 1.903967 | 1.242231532-2.918207686 | 0.0031214 | 2.70508594 | 6.16E-16 | 0.569602 | 0.000575 |
| *AC010457.1* | ENSG00000251257 | 5 | 38460925 | 38468339 | 1.108759 | 0.901452452-1.363738607 | 0.3282813 | 2.45205361 | 5.44E-06 | 1.977763 | 1.20E-09 |

| | | | | | | | | | | | |
|---|---|---|---|---|---|---|---|---|---|---|---|
| *LINC01948* | ENSG00000248727 | 5 | 56451627 | 56481946 | 0.797744 | 0.308038129-2.065965753 | 0.6416233 | 1.75676793 | 1.52E-07 | 0.819979 | 0.000108 |
| *PART1* | ENSG00000152931 | 5 | 60487713 | 60548813 | 0.865779 | 0.560608668-1.33707047 | 0.515712 | 5.95443644 | 2.00E-17 | 2.997311 | 2.11E-11 |
| *AC025176.1* | ENSG00000250081 | 5 | 65486444 | 65487048 | 1.860573 | 0.966993278-3.579891962 | 0.0629639 | 2.88213912 | 6.60E-13 | 0.706759 | 0.000908 |
| *AC112206.2* | ENSG00000249364 | 5 | 67379378 | 67806600 | 0.917307 | 0.776961625-1.083003852 | 0.3083066 | -1.5916661 | 7.63E-06 | -1.11847 | 2.11E-05 |
| *AC010280.2* | ENSG00000248884 | 5 | 68430427 | 68434481 | 1.065478 | 0.901983687-1.258607974 | 0.4555309 | -2.5940515 | 1.93E-15 | -1.6367 | 4.97E-12 |
| *LINC02200* | ENSG00000250358 | 5 | 112628436 | 112642483 | 1.013003 | 0.90094584-1.138998564 | 0.828985 | 4.288762 | 4.29E-08 | -2.42972 | 2.75E-06 |
| *C5orf66* | ENSG00000224186 | 5 | 135033280 | 135358219 | 0.815346 | 0.277373838-2.396723999 | 0.7105795 | 1.48136546 | 8.41E-08 | 0.935856 | 2.11E-06 |
| *AC113382.1* | ENSG00000246323 | 5 | 138032774 | 138037266 | 6.994351 | 0.000599472-81606.66952 | 0.6839332 | 1.36145989 | 0.000669 | 0.843698 | 0.000623 |
| *SNHG4** | ENSG00000281398 | 5 | 139274102 | 139284899 | 1.588308 | 1.096582752-2.300531942 | 0.0143756 | 2.39930387 | 1.38E-24 | 0.930488 | 1.90E-09 |
| *AC005618.1* | ENSG00000272070 | 5 | 141326210 | 141329357 | 1.813092 | 0.453239666-7.252902566 | 0.400223 | 1.9266624 | 2.60E-11 | 0.840466 | 4.63E-06 |
| *AC011352.1** | ENSG00000248362 | 5 | 147886086 | 147886878 | 2.334879 | 1.029622575-5.294813547 | 0.0423724 | 2.38168302 | 6.91E-06 | 1.17547 | 0.000574 |
| *LINC02159** | ENSG00000253417 | 5 | 160931778 | 160938626 | 1.377556 | 1.008838605-1.88103354 | 0.04387 | 4.38150239 | 2.86E-14 | 1.510857 | 3.25E-05 |
| *LINC01863* | ENSG00000253959 | 5 | 173642519 | 173658194 | 0.682755 | 0.297494715-1.566931557 | 0.3679308 | -2.3777444 | 2.63E-09 | -1.96546 | 6.88E-11 |
| *AC139491.2* | ENSG00000248596 | 5 | 176143085 | 176185155 | 1.183787 | 0.71779562-1.952299122 | 0.5086233 | 2.08526546 | 3.67E-12 | -0.79869 | 0.000307 |
| *AC139795.3* | ENSG00000272459 | 5 | 177554824 | 177555364 | 1.135884 | 0.75036849-1.719464812 | 0.5469642 | -0.716249 | 0.000311 | -0.55412 | 0.000123 |
| *AC008610.1* | ENSG00000248367 | 5 | 180293245 | 180295253 | 1.000655 | 0.8970263-1.116255087 | 0.9906357 | 1.77635029 | 5.85E-15 | 0.57338 | 0.000209 |
| *AL590004.3* | ENSG00000260604 | 6 | 3904920 | 3911979 | 1.015234 | 0.717208812-1.437098768 | 0.9320448 | 1.74021992 | 3.73E-08 | 0.977031 | 9.93E-06 |
| *AL024498.1* | ENSG00000272097 | 6 | 10743324 | 10747663 | 7.087427 | 0.684052637-73.43239029 | 0.1006631 | 2.00519605 | 1.71E-15 | 0.629818 | 2.70E-07 |
| *AL008729.1* | ENSG00000215022 | 6 | 13264861 | 13295586 | 2.016939 | 0.453376307-8.972773735 | 0.3569188 | 1.33927265 | 1.61E-13 | 0.831818 | 2.25E-13 |
| *AL023583.1* | ENSG00000272209 | 6 | 13825432 | 13826574 | 0.657575 | 0.314036407-1.376923899 | 0.2662611 | 1.1404735 | 0.000498 | 0.830217 | 0.000214 |

| | | | | | | | | | | | |
|---|---|---|---|---|---|---|---|---|---|---|---|
| *LINC01108* | ENSG00000226673 | 6 | 14280127 | 14285454 | 0.395868 | 0.072896655-2.149776272 | 0.2830878 | 2.95801792 | 3.71E-15 | 0.857836 | 0.000644 |
| *CASC15* | ENSG00000272168 | 6 | 21664185 | 22654455 | 1.99634 | 0.682481332-5.839534636 | 0.2068134 | 1.87365503 | 1.08E-10 | 1.70887 | 1.05E-18 |
| *LINC00240* | ENSG00000224843 | 6 | 26956932 | 27059749 | 1.077736 | 0.606111268-1.91633923 | 0.7987748 | 0.75282463 | 0.000148 | -0.79164 | 1.13E-08 |
| *AL009179.1* | ENSG00000281706 | 6 | 27694035 | 27713352 | 40.70408 | 0.940894772-1760.90056 | 0.0538219 | 2.25013558 | 1.82E-06 | 1.350266 | 1.70E-06 |
| *HCG18* | ENSG00000231074 | 6 | 30286690 | 30327382 | 0.94949 | 0.637945426-1.41317821 | 0.7983737 | 0.71428782 | 3.13E-11 | 0.566204 | 1.35E-14 |
| *SNHG32* | ENSG00000204387 | 6 | 31834608 | 31839766 | 1.002898 | 0.99523323-1.010621428 | 0.459753 | 1.19721687 | 1.67E-20 | 0.50976 | 7.16E-08 |
| *Z97832.2* | ENSG00000272374 | 6 | 35220370 | 35224630 | 5.039927 | 0.526174331-48.2746152 | 0.160626 | 1.63258991 | 2.79E-17 | 0.750946 | 9.13E-11 |
| *AL033384.2* | ENSG00000271218 | 6 | 53918974 | 53920788 | 2.301459 | 0.652928641-8.112238576 | 0.1947092 | 2.41325036 | 1.77E-09 | 0.80506 | 0.000156 |
| *AC019205.1* | ENSG00000229852 | 6 | 73263212 | 73301789 | 3.859058 | 0.369431553-40.31147823 | 0.2592745 | 0.90866176 | 0.00047 | 0.672289 | 0.000241 |
| *AL359715.3* | ENSG00000272129 | 6 | 80355424 | 80356859 | 1.51227 | 0.966713537-2.365706453 | 0.0700353 | -1.5681706 | 1.13E-24 | -0.55426 | 1.49E-06 |
| *Z99289.1* | ENSG00000237234 | 6 | 112154765 | 112166476 | 1.178415 | 0.076604046-18.12778342 | 0.9062876 | -1.6415336 | 1.49E-07 | -0.97998 | 3.85E-05 |
| *LINC01625* | ENSG00000238099 | 6 | 139435636 | 139474658 | 0.783295 | 0.430996908-1.42356266 | 0.4229486 | -1.5500433 | 8.71E-05 | -1.40102 | 1.84E-06 |
| *LINC00574* | ENSG00000231690 | 6 | 169790057 | 169802873 | 1.381434 | 0.936307167-2.038176828 | 0.1034576 | -0.7898913 | 4.63E-05 | -0.58642 | 4.60E-05 |
| *AL596442.2* | ENSG00000273100 | 6 | 170178681 | 170179660 | 1.068372 | 0.80371523-1.420178784 | 0.64883 | -0.8100756 | 0.000539 | -0.66697 | 2.57E-05 |
| *AC093627.7* | ENSG00000261795 | 7 | 161765 | 164972 | 0.035375 | 0.00021383-5.852108493 | 0.1998045 | 1.50879373 | 5.54E-06 | 0.736516 | 0.000676 |
| *AC091729.2* | ENSG00000226291 | 7 | 1080863 | 1082178 | 0.995047 | 0.976673482-1.013765795 | 0.6015411 | -2.6408224 | 3.45E-07 | -1.46728 | 3.02E-05 |
| *AC102953.2** | ENSG00000273230 | 7 | 1464497 | 1467522 | 1.533676 | 1.056802186-2.225734075 | 0.0244033 | 2.04986731 | 6.42E-34 | 0.643415 | 1.56E-08 |
| *AC092171.1* | ENSG00000188365 | 7 | 5419846 | 5423122 | 6.282215 | 0.085036615-464.1085914 | 0.4024927 | 1.33177888 | 8.68E-05 | 0.582562 | 0.000833 |
| *AC004160.1* | ENSG00000230333 | 7 | 11180902 | 11520175 | 1.051973 | 0.366275139-3.021354504 | 0.9250093 | -2.3517974 | 5.77E-12 | -1.55033 | 7.09E-10 |

| | | | | | | | | | | | |
|---|---|---|---|---|---|---|---|---|---|---|---|
| *AC004160.2* | ENSG00000230435 | 7 | 11406955 | 11414078 | 1.035925 | 0.633563256-1.693818411 | 0.8881133 | -2.039784 | 1.58E-06 | -1.82905 | 2.67E-08 |
| *AC005165.1** | ENSG00000223561 | 7 | 25593304 | 25751032 | 0.282441 | 0.079279861-1.006219077 | 0.0511285 | 2.67639862 | 7.25E-09 | 1.352822 | 1.97E-05 |
| *AC004870.2* | ENSG00000223829 | 7 | 46969644 | 47027481 | 1.167045 | 0.917394869-1.484631523 | 0.2084304 | 4.75017195 | 4.08E-07 | 2.832097 | 1.64E-06 |
| *AC006329.1* | ENSG00000232445 | 7 | 101308270 | 101314800 | 1.043665 | 0.986189096-1.104490771 | 0.1392015 | -1.3262956 | 5.74E-06 | -1.24806 | 3.08E-09 |
| *AC073111.1* | ENSG00000239377 | 7 | 150379854 | 150383885 | 1.800403 | 0.352213195-9.203093659 | 0.4799519 | 2.56102446 | 2.48E-13 | 0.646913 | 5.77E-05 |
| *LINC02153* | ENSG00000253199 | 8 | 20972021 | 20995119 | 167.5827 | 0.223938738-125409.1419 | 0.1293194 | -2.5538422 | 1.59E-05 | -1.9056 | 0.000251 |
| *AC124067.2* | ENSG00000253414 | 8 | 37516399 | 37521447 | 1.049653 | 0.726762202-1.515999818 | 0.796125 | 2.24638125 | 1.97E-09 | 0.756315 | 0.000848 |
| *AC138356.1* | ENSG00000183154 | 8 | 37734761 | 37737426 | 0.384073 | 0.133342959-1.106258569 | 0.0762497 | -2.3248393 | 3.49E-22 | -0.87306 | 1.85E-06 |
| *AC079089.1* | ENSG00000246528 | 8 | 69834111 | 69854971 | 1.392905 | 0.048432242-40.05976106 | 0.8466718 | 1.96729905 | 3.20E-05 | 0.975212 | 0.000688 |
| *AC011632.1* | ENSG00000253706 | 8 | 74798784 | 74866939 | 1.245402 | 0.695882348-2.228864178 | 0.4598981 | 5.64096685 | 1.61E-14 | 1.717272 | 0.000357 |
| *AC009902.3* | ENSG00000272425 | 8 | 81275399 | 81277570 | 172.3233 | 0.224353399-132359.6395 | 0.1287438 | 1.68903558 | 2.73E-05 | 0.937132 | 2.33E-05 |
| *AC009902.2** | ENSG00000254027 | 8 | 81279871 | 81281446 | 9.616832 | 2.166638544-42.68522305 | 0.0029129 | 1.78859237 | 1.19E-05 | 1.070926 | 1.75E-06 |
| *CASC8** | ENSG00000246228 | 8 | 127289817 | 127482139 | 1.554184 | 1.232891832-1.959204777 | 0.0001901 | 2.99163747 | 7.23E-10 | 1.221973 | 7.37E-05 |
| *AC105219.3* | ENSG00000254574 | 8 | 143709007 | 143713584 | 2.33E-05 | 6.31E-19-861200214.2 | 0.5033725 | 2.0709787 | 3.78E-06 | 1.231584 | 4.93E-06 |
| *AL354989.1** | ENSG00000228352 | 9 | 34084332 | 34096225 | 9.48568 | 1.116739452-80.57218029 | 0.0392918 | 1.07922536 | 0.000406 | 0.735977 | 9.15E-06 |
| *LINC01508* | ENSG00000231107 | 9 | 90300885 | 90433566 | 1.183286 | 0.971766973-1.440845238 | 0.0939468 | 2.92822415 | 0.000352 | 2.620004 | 1.51E-06 |
| *AL161729.4** | ENSG00000271659 | 9 | 95514045 | 95514520 | 2.563093 | 1.243760795-5.281919977 | 0.0107337 | 2.22439214 | 6.98E-09 | 0.700552 | 0.000659 |
| *FAM225B** | ENSG00000225684 | 9 | 113102117 | 113111543 | 7935.61 | 9.418916428-6685896.614 | 0.0089886 | 1.4456101 | 0.000239 | 0.88813 | 0.000209 |
| *MIR600HG* | ENSG00000236901 | 9 | 123109494 | 123115477 | 1.458886 | 0.909719675-2.339565888 | 0.1170433 | 1.40247855 | 4.27E-16 | 0.562749 | 5.13E-06 |
| *MIR181A2HG* | ENSG00000224020 | 9 | 124658467 | 124698631 | 0.985806 | 0.667899508-1.455029573 | 0.9426268 | 1.25708783 | 3.21E-05 | 0.88771 | 7.35E-06 |

| | | | | | | | | | | | |
|---|---|---|---|---|---|---|---|---|---|---|---|
| *AL590708.1* | ENSG00000232850 | 9 | 128128529 | 128130628 | 0.894201 | 0.13934903-5.738083122 | 0.9061464 | 3.61816328 | 2.39E-24 | 0.619374 | 0.000451 |
| *AL391056.1* | ENSG00000227619 | 9 | 129575117 | 129584556 | 1.009393 | 0.969192443-1.051261474 | 0.6520756 | 3.71488164 | 2.50E-19 | 1.035387 | 0.000447 |
| *AL355574.1* | ENSG00000238058 | 9 | 135907812 | 135916126 | 1.023471 | 0.789569081-1.326664081 | 0.860886 | 1.35520951 | 4.71E-13 | 0.556164 | 1.50E-06 |
| *SNHG7** | ENSG00000233016 | 9 | 136721366 | 136728184 | 1.031789 | 1.004086404-1.060255492 | 0.0242183 | 1.56275787 | 7.07E-23 | 0.672029 | 3.59E-09 |
| *AL355987.4** | ENSG00000273066 | 9 | 136799223 | 136810042 | 1.215544 | 1.020356226-1.448069132 | 0.0288442 | 2.22764707 | 1.33E-16 | 1.454813 | 5.99E-15 |
| *AL359878.1** | ENSG00000205740 | 10 | 971146 | 988341 | 149.7894 | 3.803908753-5898.371007 | 0.007521 | 2.4687884 | 2.88E-18 | 0.815126 | 2.54E-08 |
| *LINC00839** | ENSG00000185904 | 10 | 42475480 | 42495337 | 1.19592 | 1.027893158-1.391414827 | 0.0205527 | 1.23782867 | 3.33E-06 | 1.48671 | 2.00E-15 |
| *AL353801.1* | ENSG00000223462 | 10 | 44937508 | 44955725 | 1.111532 | 0.56214931-2.197822627 | 0.761127 | 1.93094349 | 1.09E-06 | 0.795232 | 0.000225 |
| *AL356056.2* | ENSG00000231187 | 10 | 46580708 | 46598150 | 0.967178 | 0.601175224-1.556008553 | 0.890589 | 1.18710449 | 7.31E-06 | 0.827882 | 3.10E-06 |
| *LINC02640* | ENSG00000233590 | 10 | 68233251 | 68242379 | 0.914566 | 0.617229577-1.355136298 | 0.6562111 | -1.0149771 | 0.000512 | -1.24207 | 2.73E-09 |
| *LINC02636** | ENSG00000224222 | 10 | 69994276 | 70019496 | 1.698164 | 1.06989053-2.695379068 | 0.0246677 | 2.24233862 | 6.10E-05 | 1.469202 | 1.73E-05 |
| *AC016394.2** | ENSG00000272599 | 10 | 73124573 | 73125532 | 1.558133 | 1.153255186-2.105153978 | 0.0038679 | 1.7713731 | 7.53E-30 | 0.511511 | 1.76E-07 |
| *LINC01374* | ENSG00000280560 | 10 | 89853595 | 90225443 | 0.705973 | 0.084759889-5.880114561 | 0.7475042 | 1.51878373 | 0.000643 | 1.009817 | 9.10E-05 |
| *AL133215.2** | ENSG00000273162 | 10 | 101060029 | 101061005 | 3.33686 | 1.770642583-6.288469499 | 0.0001937 | 2.65434429 | 5.50E-17 | 0.758127 | 8.74E-06 |
| *TLX1NB* | ENSG00000236311 | 10 | 101089321 | 101131126 | 2.624255 | 0.196557885-35.03656867 | 0.4656018 | 2.86269567 | 2.93E-09 | 1.072647 | 0.000187 |
| *AL121929.3* | ENSG00000287419 | 10 | 103573157 | 103583687 | 0.466464 | 0.104069034-2.09080724 | 0.3190898 | 1.64757471 | 6.98E-06 | 0.93454 | 5.88E-05 |
| *AL158212.3* | ENSG00000260917 | 10 | 112823490 | 112827726 | 1.113451 | 0.658491224-1.882747749 | 0.6884296 | 1.49928659 | 8.12E-23 | 0.541992 | 2.10E-07 |
| *AL158166.1** | ENSG00000227076 | 10 | 127934698 | 127936167 | 6.169867 | 1.456329662-26.13917543 | 0.0135003 | 1.25053313 | 0.000768 | 1.270017 | 1.65E-09 |
| *AL451069.3** | ENSG00000234311 | 10 | 132433733 | 132441484 | 1.115362 | 1.026797971-1.211565815 | 0.0096971 | 3.67085551 | 9.32E-09 | 3.653368 | 1.47E-23 |

| | | | | | | | | | | | |
|---|---|---|---|---|---|---|---|---|---|---|---|
| *AP006285.1* | ENSG00000231487 | 11 | 1662584 | 1663343 | 0.9657 | 0.749153603-1.244841093 | 0.7876134 | -1.6228157 | 8.57E-06 | -1.69138 | 1.72E-10 |
| *FAM99A* | ENSG00000205866 | 11 | 1665597 | 1667856 | 0.980112 | 0.954945704-1.005942184 | 0.1301358 | -1.9163158 | 6.92E-07 | -1.70024 | 1.04E-09 |
| *FAM99B* | ENSG00000205865 | 11 | 1683269 | 1685629 | 0.986601 | 0.941580037-1.033774618 | 0.5713461 | -1.6945256 | 3.64E-06 | -1.58802 | 1.94E-09 |
| *KCNQ1OT1* | ENSG00000269821 | 11 | 2608328 | 2699994 | 0.672949 | 0.003084864-146.800923 | 0.885376 | 1.22201328 | 1.94E-10 | 0.603345 | 1.56E-05 |
| *LINC02709* | ENSG00000245522 | 11 | 9754770 | 9759533 | 1.37335 | 0.255495197-7.382095064 | 0.7115878 | 1.46647471 | 8.06E-12 | 0.960363 | 2.78E-12 |
| *LINC00958** | ENSG00000251381 | 11 | 12961541 | 12989597 | 1.138618 | 1.00450195-1.290641218 | 0.0423349 | 3.16477662 | 2.36E-06 | 1.539094 | 0.000566 |
| *AC124798.1* | ENSG00000260196 | 11 | 17380649 | 17383531 | 1.036728 | 0.787984621-1.363992496 | 0.7966491 | 1.06498858 | 0.000198 | 0.733801 | 0.000521 |
| *AC004923.4* | ENSG00000255306 | 11 | 68024809 | 68030461 | 1.598139 | 0.595643108-4.287882583 | 0.3518245 | 1.48339253 | 4.79E-10 | 0.676031 | 7.68E-07 |
| *AP002761.3* | ENSG00000257038 | 11 | 73307235 | 73309361 | 1.060254 | 0.645045652-1.742726273 | 0.8175018 | -0.8475016 | 0.000305 | 0.778653 | 7.31E-06 |
| *AP001527.2* | ENSG00000277459 | 11 | 102109827 | 102110457 | 1.484561 | 0.691719268-3.186148569 | 0.3105621 | 1.05999066 | 1.08E-05 | 0.828301 | 5.13E-09 |
| *COLCA1* | ENSG00000196167 | 11 | 111290787 | 111305498 | 0.926763 | 0.643748747-1.334200995 | 0.6824717 | 1.80979546 | 4.27E-13 | 0.667223 | 0.000443 |
| *AP000757.2* | ENSG00000254844 | 11 | 117818443 | 117821992 | 0.920911 | 0.768463781-1.103599676 | 0.3722121 | 2.53433615 | 4.36E-06 | 2.180406 | 1.86E-08 |
| *AP000866.2* | ENSG00000250073 | 11 | 124759129 | 124766119 | 1.806967 | 0.733627765-4.450660407 | 0.1982854 | 1.1384025 | 3.09E-08 | 0.562962 | 2.41E-05 |
| *AP000842.3* | ENSG00000260209 | 11 | 125873031 | 125874528 | 27.01372 | 0.357952749-2038.652657 | 0.1351085 | 2.20217267 | 9.99E-07 | 0.963432 | 0.000411 |
| *GSEC* | ENSG00000280832 | 11 | 126340889 | 126355587 | 1.462502 | 0.831616859-2.57199346 | 0.1868969 | 1.32322327 | 2.27E-15 | 0.500473 | 3.84E-05 |
| *LINC00942* | ENSG00000249628 | 12 | 1500525 | 1504424 | 1.007122 | 0.979718445-1.035292555 | 0.6141088 | 6.14189406 | 1.21E-24 | 2.444532 | 2.21E-10 |
| *LINC01559* | ENSG00000180861 | 12 | 13371089 | 13387167 | 0.870302 | 0.526529299-1.438525443 | 0.5879625 | 2.20461732 | 0.000217 | 1.998637 | 5.10E-07 |
| *AC008250.2* | ENSG00000275567 | 12 | 21828360 | 21828564 | 0.697423 | 0.441260814-1.102292648 | 0.1228401 | -1.2825603 | 2.23E-06 | -0.79521 | 0.000152 |
| *LINC00941* | ENSG00000235884 | 12 | 30755167 | 30802602 | 1.305557 | 0.668113731-2.55118271 | 0.4353537 | 2.5541883 | 5.25E-12 | 1.729912 | 3.25E-12 |
| *AC048344.4* | ENSG00000277342 | 12 | 32109076 | 32109602 | 4.124124 | 0.691454361-24.59800267 | 0.1199401 | 1.44027556 | 0.000268 | 0.871317 | 0.000118 |

| | | | | | | | | | | | |
|---|---|---|---|---|---|---|---|---|---|---|---|
| *AC026356.2* | ENSG00000276115 | 12 | 32352349 | 32354144 | 3.749964 | 0.348190734-40.38656697 | 0.2757287 | 1.40156622 | 1.91E-08 | 0.701977 | 1.52E-06 |
| *LINC02156** | ENSG00000257906 | 12 | 47377638 | 47420192 | 1.623931 | 1.048854076-2.514317939 | 0.0297188 | -1.7391067 | 2.00E-06 | -1.07529 | 0.000162 |
| *AC125611.4** | ENSG00000258334 | 12 | 49292631 | 49324576 | 73791408 | 10.43215663-5.2196E+14 | 0.0243625 | 1.62417633 | 0.000124 | 0.927172 | 0.000266 |
| *LINC02381* | ENSG00000250742 | 12 | 54126082 | 54147485 | 1.013801 | 0.96890885-1.06077259 | 0.5530869 | 1.14363594 | 0.000122 | 1.501522 | 1.44E-12 |
| *AC078778.1* | ENSG00000258344 | 12 | 54276631 | 54345083 | 3.664544 | 0.174861795-76.79712074 | 0.4028012 | 1.40624837 | 0.00026 | 0.723449 | 0.00087 |
| *LINC02421* | ENSG00000255970 | 12 | 67709047 | 67729475 | 35.53913 | 0.489085568-2582.431666 | 0.1024926 | 2.05094736 | 1.84E-06 | 0.864295 | 0.000462 |
| *AC020656.2* | ENSG00000274979 | 12 | 69326574 | 69331882 | 1.163856 | 0.670828377-2.019234281 | 0.5893571 | 1.46660323 | 1.11E-06 | 0.848893 | 0.00012 |
| *AC083809.1* | ENSG00000277247 | 12 | 70570969 | 70571440 | 0.997811 | 0.985592735-1.010181046 | 0.7274064 | 3.02099509 | 2.87E-08 | 2.305571 | 1.61E-09 |
| *LINC01619* | ENSG00000257242 | 12 | 91984976 | 92142914 | 0.461668 | 0.109466981-1.947049095 | 0.2925414 | 0.89670485 | 0.000499 | 0.629345 | 0.000747 |
| *LINC02413* | ENSG00000257345 | 12 | 92999218 | 93019820 | 0.669431 | 0.365519547-1.226029001 | 0.193631 | 4.11314246 | 1.70E-09 | 3.553029 | 3.46E-17 |
| *AC007298.2* | ENSG00000257878 | 12 | 95996521 | 96011489 | 1.043299 | 0.782229401-1.391500413 | 0.7729858 | -1.6686035 | 4.70E-08 | -1.00553 | 1.29E-05 |
| *AC089983.1* | ENSG00000257732 | 12 | 104262314 | 104280722 | 0.92871 | 0.346021299-2.492626836 | 0.8832725 | 2.90907004 | 5.03E-08 | 1.106687 | 0.000854 |
| *AC026368.1* | ENSG00000277840 | 12 | 117002463 | 117003152 | 1.170557 | 0.737029711-1.859089981 | 0.5046393 | 2.06729811 | 2.15E-08 | 1.564566 | 2.25E-13 |
| *AC069234.5* | ENSG00000277423 | 12 | 120703867 | 120704282 | 1.744336 | 0.740120422-4.111100344 | 0.2033869 | 1.28126229 | 7.66E-06 | 0.6732 | 5.49E-05 |
| *AC156455.1* | ENSG00000256546 | 12 | 122063306 | 122068616 | 0.68986 | 0.253758509-1.87543125 | 0.4668629 | 0.8597889 | 0.000495 | 0.649389 | 0.000256 |
| *AC145423.2* | ENSG00000274427 | 12 | 123515275 | 123515513 | 1.1503 | 0.978249259-1.352610262 | 0.0902786 | 1.69439898 | 1.22E-08 | 0.627829 | 0.000484 |
| *AC135586.2* | ENSG00000256632 | 12 | 132688049 | 132714912 | 0.590447 | 0.189306745-1.841603615 | 0.3639733 | -1.1399306 | 9.69E-10 | -0.69941 | 6.63E-07 |
| *AL161772.1* | ENSG00000278291 | 13 | 20699307 | 20703718 | 1.293532 | 0.943130812-1.774116748 | 0.1103268 | 1.91122029 | 2.72E-08 | 0.882139 | 7.17E-05 |
| *LINC00327* | ENSG00000232977 | 13 | 23465776 | 23487712 | 0.002101 | 2.59E-06-1.705584496 | 0.0712689 | 1.46241385 | 0.00032 | 1.121082 | 9.29E-06 |

| | | | | | | | | | | | |
|---|---|---|---|---|---|---|---|---|---|---|---|
| *AL161891.1* | ENSG00000276672 | 13 | 33846190 | 33850825 | 0.930539 | 0.122570429-7.064536009 | 0.944506 | 1.18029085 | 2.89E-10 | 0.551651 | 3.41E-05 |
| *AL356740.3* | ENSG00000269376 | 13 | 113009671 | 113010319 | 0.842345 | 0.439807208-1.613310118 | 0.6048471 | 2.40008681 | 7.63E-05 | 1.341036 | 0.000625 |
| *AL442125.1* * | ENSG00000276248 | 13 | 113527260 | 113530621 | 30.38451 | 1.147030882-804.876473 | 0.04115 | 1.07840104 | 1.41E-06 | 0.513358 | 0.000514 |
| *AL356019.2* | ENSG00000258768 | 14 | 20260480 | 20264308 | 2.016009 | 0.547361931-7.42523774 | 0.2918824 | 1.03093847 | 5.55E-08 | 0.683662 | 4.34E-08 |
| *AL161668.4* | ENSG00000258604 | 14 | 21024172 | 21029271 | 0.999514 | 0.924415979-1.080711789 | 0.9902574 | -1.9174954 | 3.03E-13 | -0.78438 | 8.96E-05 |
| *AL121790.1* | ENSG00000258414 | 14 | 37564047 | 37579125 | 5.262302 | 0.880392549-31.45395258 | 0.0687101 | -1.336481 | 2.96E-05 | -1.07683 | 7.15E-06 |
| *AL163953.1* | ENSG00000225680 | 14 | 53599832 | 53614026 | 0.809417 | 0.560204369-1.169494026 | 0.2601251 | 3.80749967 | 1.06E-17 | 1.27787 | 1.13E-06 |
| *AL359232.1* | ENSG00000258561 | 14 | 66212810 | 66509394 | 2.497994 | 0.290768284-21.46030284 | 0.404117 | -0.8077796 | 0.000104 | -0.68539 | 8.07E-06 |
| *LINC01269* | ENSG00000258689 | 14 | 70698698 | 70712153 | 1.001789 | 0.890794206-1.126614106 | 0.9761996 | 3.15400984 | 2.86E-11 | 1.006475 | 0.000346 |
| *AC005225.2* | ENSG00000258603 | 14 | 73555115 | 73633941 | 1.194652 | 0.890522556-1.602648162 | 0.2354334 | -0.7212067 | 0.000101 | -0.54942 | 6.21E-05 |
| *AF111167.2* | ENSG00000259319 | 14 | 75423683 | 75427741 | 1.299169 | 0.633018163-2.66633834 | 0.4755549 | -0.7772317 | 0.000201 | -0.57689 | 0.000238 |
| *AL133279.1* * | ENSG00000258538 | 14 | 88175391 | 88180198 | 6.743691 | 1.318714991-34.48612528 | 0.0218925 | -1.1452646 | 0.000301 | -1.30567 | 3.38E-08 |
| *AL137230.2* | ENSG00000259053 | 14 | 89412312 | 89954659 | 1.856886 | 0.657302145-5.245723674 | 0.2427908 | 1.40054534 | 1.28E-08 | -0.70451 | 2.45E-05 |
| *LINC02321* | ENSG00000258884 | 14 | 90820064 | 90828199 | 1.136368 | 0.952569606-1.355630612 | 0.1555635 | 0.95416931 | 9.07E-05 | -0.62995 | 0.000261 |
| *AL133467.1* | ENSG00000258572 | 14 | 95516136 | 95517911 | 0.999698 | 0.545858114-1.830873156 | 0.9992205 | -0.931784 | 7.87E-05 | -0.65378 | 0.000203 |
| *AL355102.4* | ENSG00000258793 | 14 | 96259411 | 96268624 | 0.995061 | 0.943789318-1.049117334 | 0.8544411 | 2.04279622 | 9.28E-10 | 0.99145 | 6.72E-05 |
| *MEG3* | ENSG00000214548 | 14 | 100779410 | 100861031 | 1.007153 | 0.976852758-1.038393422 | 0.6474352 | 1.26871427 | 0.00024 | 1.143751 | 3.83E-06 |
| *AL161669.3* * | ENSG00000278071 | 14 | 103208100 | 103208876 | 1.250841 | 1.113895204-1.40462346 | 0.0001548 | 1.37509844 | 3.95E-11 | -0.52927 | 0.000516 |
| *AC026495.1* | ENSG00000258654 | 15 | 20344736 | 20359166 | 2.616509 | 0.918647841-7.452386834 | 0.071691 | 2.10808558 | 1.22E-06 | -1.04407 | 0.000174 |
| *SNHG14* | ENSG00000224078 | 15 | 24978583 | 25420336 | 1.063529 | 0.835984593-1.353007676 | 0.6160511 | 0.82582162 | 0.000715 | 0.970592 | 2.22E-08 |

| | | | | | | | | | | | |
|---|---|---|---|---|---|---|---|---|---|---|---|
| *AC124312.3* | ENSG00000261069 | 15 | 25087661 | 25088896 | 1.216946 | 0.599597236-2.469921598 | 0.5866724 | 1.29933652 | 0.000495 | 1.15505 | 3.96E-06 |
| *AC091057.1* | ENSG00000187951 | 15 | 30658717 | 30772993 | 1.889094 | 0.797320259-4.475839341 | 0.1483679 | 2.11639504 | 4.72E-32 | 0.747985 | 7.61E-10 |
| *AC078909.2* | ENSG00000274444 | 15 | 37109587 | 37109984 | 0.887051 | 0.639251476-1.230906384 | 0.473344 | 3.17160918 | 7.34E-16 | 0.799809 | 0.000101 |
| *AC020658.4* * | ENSG00000273786 | 15 | 40226386 | 40227069 | 17.63585 | 1.784120013-174.3287218 | 0.0140792 | 2.50275903 | 6.37E-09 | 0.990753 | 6.43E-05 |
| *AC020661.1* | ENSG00000251161 | 15 | 40906811 | 40910337 | 1.997636 | 0.446962568-8.928151412 | 0.3650343 | 1.1643713 | 1.77E-06 | 0.715684 | 8.41E-07 |
| *AC025580.1* * | ENSG00000259342 | 15 | 45430652 | 45441808 | 1.522323 | 1.055371992-2.195877158 | 0.0245573 | 2.12096248 | 2.21E-05 | 1.74097 | 6.08E-08 |
| *AC104574.2* | ENSG00000259616 | 15 | 61298791 | 61635449 | 2.670028 | 0.683187165-10.43498898 | 0.1579075 | 1.81756724 | 0.000393 | -1.15764 | 0.000173 |
| *AC103740.1* | ENSG00000259370 | 15 | 62827675 | 62884034 | 1.246237 | 0.731440212-2.123355392 | 0.4181332 | -1.3783106 | 2.01E-07 | -0.82876 | 3.08E-05 |
| *AC026992.2* | ENSG00000261634 | 15 | 69278328 | 69298795 | 2.67331 | 0.409607699-17.44739337 | 0.3042334 | 2.36612169 | 1.03E-07 | 1.260091 | 1.37E-05 |
| *LINC02259* | ENSG00000259783 | 15 | 72608481 | 72636962 | 0.451781 | 0.110955511-1.839529874 | 0.2673707 | -0.9021792 | 0.000156 | -0.75711 | 2.03E-05 |
| *LINC01579* | ENSG00000258754 | 15 | 93718542 | 94070898 | 6.229941 | 0.010542927-3681.345853 | 0.5742232 | 2.89710669 | 2.16E-05 | 1.649582 | 0.000213 |
| *Z92544.1* | ENSG00000260394 | 16 | 678504 | 679777 | 1.114447 | 0.205715089-6.03743751 | 0.8999727 | 0.88737568 | 1.58E-06 | 0.748451 | 3.94E-11 |
| *AL031705.1* | ENSG00000260646 | 16 | 1512979 | 1514675 | 2.984958 | 0.423855728-21.0212492 | 0.2721709 | 1.87949453 | 2.11E-07 | 0.671387 | 0.00028 |
| *AL031722.1* | ENSG00000260541 | 16 | 1841020 | 1843547 | 0.770766 | 0.579646452-1.024900006 | 0.0733251 | -1.0688748 | 3.76E-07 | -0.71215 | 6.56E-06 |
| *AC012676.1* * | ENSG00000262712 | 16 | 4335870 | 4337818 | 1.93953 | 1.010689126-3.721993596 | 0.0463782 | 1.40213834 | 1.18E-12 | 0.60529 | 8.07E-06 |
| *AC009093.1* | ENSG00000259807 | 16 | 29215385 | 29221040 | 1.719608 | 0.27185036-10.87749743 | 0.5646153 | 1.1170625 | 3.05E-05 | 0.637736 | 0.00021 |
| *AC009093.5* | ENSG00000273582 | 16 | 29225594 | 29226348 | 1.01166 | 0.828159702-1.235818877 | 0.9096169 | -1.3815593 | 6.39E-05 | -1.34534 | 7.00E-08 |
| *AC135048.1* | ENSG00000261487 | 16 | 30948386 | 30956511 | 1.071438 | 0.694042771-1.654045869 | 0.7554562 | -0.7853643 | 0.000187 | -0.61066 | 9.07E-05 |
| *AC007344.1* | ENSG00000260850 | 16 | 51354456 | 51525157 | 2.918321 | 0.700572907-12.1566169 | 0.1412496 | -1.5227419 | 0.000276 | -1.36633 | 3.48E-05 |

| | | | | | | | | | | | |
|---|---|---|---|---|---|---|---|---|---|---|---|
| *AC040168.1* | ENSG00000246379 | 16 | 55984009 | 56191118 | 0.312688 | 0.018605631-5.25507556 | 0.4193794 | -1.3331888 | 0.000594 | -1.20888 | 4.33E-05 |
| *AC026461.2* | ENSG00000259923 | 16 | 56648881 | 56652636 | 0.956705 | 0.733521364-1.247794578 | 0.7439947 | -3.0285505 | 1.68E-11 | -1.90587 | 1.17E-07 |
| *AC027682.6* | ENSG00000276075 | 16 | 67517862 | 67528675 | 1.048944 | 0.994186836-1.106717829 | 0.0806661 | -0.6849061 | 0.000704 | -0.65577 | 6.21E-06 |
| *LINC01572* | ENSG00000261008 | 16 | 72236281 | 72665014 | 2.359065 | 0.04111062-135.3710139 | 0.677867 | 2.20792081 | 1.04E-14 | 0.585365 | 0.000882 |
| *AC009159.3* | ENSG00000278058 | 16 | 79605802 | 79606605 | 0.965878 | 0.750918437-1.242372238 | 0.7869289 | -1.2816562 | 3.34E-13 | -0.57261 | 1.41E-05 |
| *AC026469.1* | ENSG00000275088 | 16 | 85142696 | 85146001 | 1.299413 | 0.565770686-2.984378878 | 0.5369835 | -1.2439741 | 4.15E-05 | -0.90855 | 1.99E-05 |
| *LINC02188* | ENSG00000261175 | 16 | 86710122 | 86742083 | 1.128169 | 0.810120708-1.571080385 | 0.4753961 | 2.75062146 | 1.85E-09 | 2.158765 | 9.31E-14 |
| *AC134312.6* | ENSG00000261816 | 16 | 88191486 | 88193667 | 2.454265 | 0.482379859-12.48687706 | 0.2794009 | -1.4082742 | 0.000206 | -1.10797 | 0.000207 |
| *AC092384.2* | ENSG00000259881 | 16 | 88881038 | 88887136 | 1.045367 | 0.728734481-1.49957464 | 0.8095481 | -2.3076925 | 2.33E-09 | -1.4274 | 8.44E-07 |
| *AC137932.1* | ENSG00000260279 | 16 | 89296128 | 89298317 | 0.921493 | 0.666785823-1.273495026 | 0.6203761 | 0.92358005 | 5.88E-06 | -0.52307 | 0.000283 |
| *AC129507.2* | ENSG00000262294 | 17 | 352638 | 357581 | 1.128267 | 0.938623299-1.356227845 | 0.1986718 | -1.2388348 | 1.19E-08 | -0.77273 | 1.39E-06 |
| *AC099684.1* | ENSG00000228133 | 17 | 2017716 | 2020706 | 1.099259 | 0.7742039-1.560791457 | 0.5967268 | -1.0805397 | 6.90E-05 | -0.83943 | 2.54E-05 |
| *AC027796.4* | ENSG00000262903 | 17 | 3655621 | 3658092 | 0.95334 | 0.708826409-1.282199549 | 0.7519909 | 1.80058327 | 4.04E-15 | 0.590546 | 0.000146 |
| *AC087742.1* * | ENSG00000274363 | 17 | 4267691 | 4268430 | 4.012918 | 1.066637166-15.0974613 | 0.0398414 | 1.83554718 | 3.10E-08 | 0.621557 | 0.000426 |
| *AC015908.3* | ENSG00000264016 | 17 | 10741257 | 10769089 | 0.966821 | 0.78183101-1.195582161 | 0.7555002 | -1.3734152 | 1.24E-13 | -0.86558 | 7.79E-11 |
| *AC005224.2* | ENSG00000266378 | 17 | 14327335 | 14329474 | 3.35267 | 0.170014747-66.11423124 | 0.4264796 | -1.2863771 | 1.95E-05 | -0.79845 | 0.000551 |
| *AC093484.4* | ENSG00000266651 | 17 | 16440479 | 16440952 | 1.57454 | 0.921221617-2.691182956 | 0.0969288 | 1.00831066 | 4.74E-05 | 0.539502 | 0.000717 |
| *AC005722.4* | ENSG00000264785 | 17 | 19737682 | 19738542 | 0.949212 | 0.764930528-1.17788925 | 0.6360027 | 3.87357108 | 4.76E-08 | 1.690462 | 0.000158 |
| *AC005730.2* | ENSG00000261033 | 17 | 20008051 | 20009234 | 0.698059 | 0.26701214-1.824958302 | 0.4634992 | 1.89478792 | 0.000637 | 1.249122 | 0.00023 |
| *AC006449.2* | ENSG00000275532 | 17 | 38703480 | 38706261 | 0.982839 | 0.378092922-2.554853786 | 0.9716694 | 1.115684 | 1.28E-06 | 0.556458 | 3.28E-05 |

| | | | | | | | | | | | |
|---|---|---|---|---|---|---|---|---|---|---|---|
| *AC060780.1* | ENSG00000267002 | 17 | 43148368 | 43171037 | 0.981741 | 0.889934378-1.083018426 | 0.7129671 | 0.71739411 | 9.38E-06 | 0.587314 | 1.09E-07 |
| *AC091133.3* | ENSG00000250838 | 17 | 48995266 | 48997492 | 1.284977 | 0.770155292-2.143939222 | 0.3370402 | 3.04636409 | 0.000924 | 2.145606 | 0.000277 |
| *AC011921.1* | ENSG00000259349 | 17 | 60526293 | 60550798 | 2.236211 | 0.787725515-6.348202519 | 0.1305967 | 2.36305006 | 8.24E-18 | 0.62128 | 1.87E-06 |
| *LINC01993* | ENSG00000204277 | 17 | 78261349 | 78278492 | 0.762954 | 0.112380092-5.179729572 | 0.7818833 | 1.47465246 | 0.000529 | 1.190879 | 4.43E-05 |
| *LINC01978* | ENSG00000262188 | 17 | 79919357 | 79925999 | 0.586805 | 0.15792652-2.180382359 | 0.4260394 | 1.11230313 | 0.000203 | 1.019068 | 2.16E-07 |
| *AC116025.2** | ENSG00000262172 | 17 | 79991944 | 79992841 | 2.414076 | 1.446456208-4.028994367 | 0.0007451 | 3.3053428 | 2.58E-19 | 0.7116 | 6.52E-05 |
| *AC132872.2* | ENSG00000264548 | 17 | 82214227 | 82217352 | 2.77092 | 0.152215943-50.44147842 | 0.4911858 | 2.61614702 | 5.64E-19 | 0.574769 | 0.000203 |
| *AP002478.1* | ENSG00000266401 | 18 | 3653030 | 3656282 | 1.174257 | 0.932000208-1.479484572 | 0.1730077 | 4.44834053 | 5.42E-12 | 1.903118 | 3.12E-06 |
| *AC006557.1** | ENSG00000267150 | 18 | 14089935 | 14091019 | 23.4933 | 1.033813445-533.8827443 | 0.0476102 | 2.12627524 | 1.41E-07 | 1.103503 | 4.09E-07 |
| *AC023983.2* | ENSG00000273321 | 18 | 24402153 | 24402714 | 0.809306 | 0.214969062-3.046839901 | 0.7544259 | 1.67719 | 0.000377 | 0.964242 | 0.000781 |
| *PCAT18* | ENSG00000265369 | 18 | 26687621 | 26703638 | 3.915832 | 0.054647892-280.5916811 | 0.5311287 | -1.535594 | 9.81E-06 | -0.92732 | 0.000801 |
| *AC011815.1* | ENSG00000268573 | 18 | 35280867 | 35290201 | 0.993159 | 0.33477316-2.946369535 | 0.990129 | 1.2025434 | 5.02E-10 | 0.627031 | 2.85E-06 |
| *LINC01901* | ENSG00000267413 | 18 | 39841174 | 39924840 | 1.696693 | 0.570654783-5.044674007 | 0.3416338 | 3.7091014 | 6.79E-09 | 1.446907 | 0.000369 |
| *AC090227.3* | ENSG00000267674 | 18 | 49739823 | 49742063 | 1.019597 | 0.367846572-2.8261166 | 0.970238 | -2.6166873 | 2.92E-09 | -1.26915 | 0.00045 |
| *AC090409.1* | ENSG00000267279 | 18 | 61585746 | 61607006 | 1.545869 | 0.787262208-3.035469113 | 0.2057989 | 1.48575096 | 2.89E-09 | 0.580217 | 0.000173 |
| *AC036176.1* | ENSG00000267390 | 18 | 63367328 | 63381629 | 1.014034 | 0.91211801-1.12733817 | 0.796499 | -0.9415269 | 4.49E-08 | -0.7047 | 1.22E-08 |
| *AC023090.1* | ENSG00000267015 | 18 | 79340258 | 79343972 | 1.064962 | 0.983861208-1.152748158 | 0.1193832 | 5.12578248 | 3.79E-17 | 1.849857 | 5.25E-07 |
| *RBFADN** | ENSG00000261126 | 18 | 80046900 | 80095482 | 2.829557 | 1.179592987-6.787420442 | 0.0198084 | 0.90206553 | 4.60E-07 | 0.515232 | 4.49E-05 |
| *AC006273.1* | ENSG00000272473 | 19 | 781002 | 781862 | 0.977235 | 0.838222365-1.13930212 | 0.7686508 | 1.2934012 | 5.72E-05 | 1.119675 | 8.51E-08 |

| | | | | | | | | | | | |
|---|---|---|---|---|---|---|---|---|---|---|---|
| *LINC01836* | ENSG00000267530 | 19 | 782755 | 786092 | 1.011853 | 0.882512459-1.160148807 | 0.8659069 | 1.16062096 | 3.36E-05 | 0.957921 | 1.55E-06 |
| *LINC01775* | ENSG00000267201 | 19 | 2458935 | 2462185 | 3.228288 | 0.560640788-18.58916756 | 0.1894891 | 2.38656083 | 3.32E-10 | 0.68266 | 0.000923 |
| *AC006538.1** | ENSG00000261342 | 19 | 2727743 | 2729327 | 1.913743 | 1.144497749-3.200015373 | 0.0133417 | 2.388644 | 1.52E-18 | 0.667762 | 0.000206 |
| *AC010336.2* | ENSG00000260500 | 19 | 7918652 | 7919157 | 0.847802 | 0.598016524-1.201920443 | 0.3538428 | -1.1778809 | 5.17E-06 | -0.97959 | 2.21E-07 |
| *AC011447.3* | ENSG00000267383 | 19 | 20122569 | 20321305 | 3.475246 | 0.742837592-16.25837833 | 0.1135722 | 1.52168262 | 3.77E-08 | 0.903128 | 4.13E-07 |
| *AC010615.2** | ENSG00000268119 | 19 | 21444241 | 21463908 | 1.769526 | 1.061586071-2.94957148 | 0.0285812 | 2.46171478 | 4.49E-14 | 0.960384 | 3.19E-05 |
| *AC006504.1* | ENSG00000261770 | 19 | 27757184 | 27760849 | 6.278483 | 0.467432844-84.331576 | 0.1657007 | 0.80812082 | 0.000126 | 0.70049 | 2.37E-07 |
| *AC006504.7** | ENSG00000281468 | 19 | 27802838 | 27803472 | 1.365759 | 1.06328862-1.754271444 | 0.0146706 | 1.146101 | 5.12E-10 | 0.620265 | 3.62E-07 |
| *AC007773.1* | ENSG00000267213 | 19 | 32390050 | 32405560 | 1.030051 | 0.90950929-1.166569332 | 0.6410198 | 1.72150138 | 9.34E-15 | 0.505729 | 0.000533 |
| *AC020907.4* | ENSG00000271032 | 19 | 35014961 | 35025335 | 1.326602 | 0.993434229-1.771503494 | 0.0554516 | 1.70683218 | 4.22E-10 | 0.67594 | 4.46E-05 |
| *AC092296.2* | ENSG00000267053 | 19 | 36304564 | 36321274 | 4.333985 | 0.675250092-27.81699301 | 0.1221043 | 2.49501786 | 7.70E-12 | 0.973084 | 4.12E-06 |
| *LINC00665* | ENSG00000232677 | 19 | 36313067 | 36331770 | 1.084013 | 0.980084889-1.198960943 | 0.1167017 | 2.79937129 | 5.30E-25 | 0.994308 | 7.15E-07 |
| *AC074138.1* | ENSG00000276071 | 19 | 36668102 | 36669404 | 1.080642 | 0.106591945-10.95567664 | 0.9476771 | 1.98880199 | 9.27E-11 | 0.848545 | 3.13E-08 |
| *AC020928.1* | ENSG00000267260 | 19 | 36773112 | 36777078 | 1.12767 | 0.798534196-1.592467756 | 0.4950238 | 2.65442072 | 4.91E-11 | 1.582744 | 1.45E-10 |
| *AC016590.1* | ENSG00000267605 | 19 | 37235507 | 37304395 | 2.888348 | 0.380428179-21.92937427 | 0.3051113 | 2.30444156 | 8.22E-09 | 1.077744 | 5.64E-07 |
| *LINC01535* | ENSG00000226686 | 19 | 37251885 | 37265535 | 0.971267 | 0.484217739-1.948213139 | 0.9345744 | 3.5382071 | 6.10E-21 | 1.396492 | 7.16E-10 |
| *AC022148.1* | ENSG00000180458 | 19 | 37545470 | 37549171 | 8.719968 | 0.234454813-324.3176754 | 0.2404817 | 2.0560834 | 2.91E-05 | 1.046739 | 0.000619 |
| *AC005393.1* | ENSG00000276445 | 19 | 39812972 | 39813555 | 1.047398 | 0.784406001-1.398563475 | 0.7535892 | 2.24074271 | 6.03E-15 | 0.840169 | 3.36E-08 |
| *LINC01480* | ENSG00000270164 | 19 | 41535183 | 41536904 | 0.976899 | 0.905191187-1.054288019 | 0.5479357 | 2.25207697 | 8.55E-12 | 0.956913 | 3.72E-05 |
| *AC006213.3* | ENSG00000267191 | 19 | 43901909 | 43927767 | 1.06319 | 0.822875964-1.373685129 | 0.6392781 | 1.95081959 | 6.49E-08 | 0.964898 | 7.23E-05 |

| | | | | | | | | | | | |
|---|---|---|---|---|---|---|---|---|---|---|---|
| *AC006213.4* * | ENSG00000277806 | 19 | 43976815 | 43977448 | 1.220888 | 1.010522772-1.475045459 | 0.0385974 | 1.05096049 | 3.76E-07 | 0.510928 | 0.000523 |
| *AC006213.5* | ENSG00000278492 | 19 | 43978376 | 43978663 | 1.420099 | 0.948638131-2.125870756 | 0.0884158 | 1.16888033 | 6.19E-05 | 0.649655 | 0.000537 |
| *AC021092.1* | ENSG00000186019 | 19 | 44103007 | 44113183 | 1.404163 | 0.278780441-7.072492649 | 0.6807101 | 1.88575675 | 3.90E-09 | 1.329821 | 1.98E-12 |
| *AC022150.4* | ENSG00000269825 | 19 | 52650437 | 52653284 | 0.552359 | 0.195698299-1.559034536 | 0.2622164 | 1.5611896 | 7.48E-07 | 0.826727 | 0.000264 |
| *AC005261.2* | ENSG00000268678 | 19 | 57350848 | 57352012 | 1.072809 | 0.990760595-1.161652236 | 0.0833981 | 1.01375126 | 0.000936 | 0.862472 | 2.46E-06 |
| *AC004076.2* | ENSG00000276449 | 19 | 57449689 | 57453011 | 3.132852 | 0.727111495-13.49828904 | 0.125438 | 1.37994788 | 1.97E-12 | 0.691577 | 3.16E-08 |
| *AC008969.1* | ENSG00000176593 | 19 | 58002061 | 58011232 | 1.783193 | 0.606604383-5.241931244 | 0.2930977 | 0.80572043 | 1.75E-06 | 0.617924 | 1.98E-07 |
| *AC020915.2* | ENSG00000268516 | 19 | 58257270 | 58278808 | 0.970351 | 0.530924298-1.773476373 | 0.9220753 | 1.52346594 | 1.00E-09 | 0.637698 | 1.53E-05 |
| *LINC02560* | ENSG00000268307 | 19 | 58400221 | 58400679 | 1.178951 | 0.335066508-4.148211046 | 0.7975834 | 1.98515361 | 4.46E-05 | 1.178238 | 5.27E-05 |
| *AL031665.1* | ENSG00000274269 | 20 | 1186092 | 1207036 | 1.363761 | 0.952215697-1.953175403 | 0.0904931 | 2.1172476 | 3.15E-06 | 1.013045 | 0.00011 |
| *AL117335.1* | ENSG00000276649 | 20 | 1888128 | 1894374 | 1.185636 | 0.822491936-1.709114085 | 0.3614426 | 1.57351256 | 1.43E-09 | 1.258552 | 1.60E-13 |
| *AL109976.1* | ENSG00000277287 | 20 | 3239705 | 3245382 | 1.129793 | 0.3364849-3.793429937 | 0.8434598 | 2.08962153 | 2.45E-11 | 0.768949 | 0.000203 |
| *AL118505.1* | ENSG00000278192 | 20 | 6065966 | 6067897 | 0.960772 | 0.349442779-2.641584713 | 0.9381857 | 2.54414886 | 3.69E-10 | 1.415258 | 5.48E-08 |
| *AL354824.2* | ENSG00000232448 | 20 | 10172701 | 10186740 | 4.272606 | 0.960366159-19.00854752 | 0.0565384 | 1.58853823 | 0.000618 | 0.968401 | 0.000483 |
| *AL121772.3* | ENSG00000276952 | 20 | 25284915 | 25285588 | 1.224003 | 0.465279955-3.219963377 | 0.6821154 | 1.6440464 | 1.06E-11 | 0.521855 | 0.000187 |
| *FAM182B* | ENSG00000175170 | 20 | 25762384 | 25868225 | 1.155807 | 0.331285313-4.032442803 | 0.8203325 | 2.11292003 | 9.91E-14 | 0.845006 | 3.22E-06 |
| *AL117382.2* | ENSG00000233376 | 20 | 44389624 | 44391537 | 0.686524 | 0.463908278-1.015967741 | 0.0600062 | 1.07853412 | 0.000344 | -0.85057 | 7.14E-05 |
| *AL031055.1* | ENSG00000271784 | 20 | 46689659 | 46690289 | 1.203801 | 0.728821717-1.988327591 | 0.4687828 | -0.9299595 | 5.51E-07 | -0.74441 | 3.27E-08 |
| *LINC01754* | ENSG00000224565 | 20 | 47391925 | 47412327 | 0.92184 | 0.597667672-1.421842317 | 0.7128032 | 1.87071879 | 1.47E-06 | 1.033594 | 7.59E-05 |

| | | | | | | | | | | | |
|---|---|---|---|---|---|---|---|---|---|---|---|
| *LINC00494* | ENSG00000235621 | 20 | 48359884 | 48370636 | 1.109784 | 0.94350233-1.305370691 | 0.208482 | 2.93353487 | 6.36E-09 | 1.868132 | 3.87E-07 |
| *ZFAS1* | ENSG00000177410 | 20 | 49278178 | 49299600 | 1.003901 | 0.994257892-1.013636833 | 0.4292042 | 0.97839378 | 6.79E-10 | 0.500773 | 2.18E-05 |
| *LINC01711* | ENSG00000268941 | 20 | 58634772 | 58635738 | 2.283475 | 0.803670351-6.48805799 | 0.1212038 | 2.64774717 | 2.87E-06 | 1.403531 | 5.82E-05 |
| *AJ009632.2* | ENSG00000229425 | 21 | 15368966 | 15627342 | 0.945147 | 0.857639783-1.041581831 | 0.2550836 | 1.52299589 | 5.34E-05 | -1.09558 | 4.92E-05 |
| *MIR99AHG* | ENSG00000215386 | 21 | 15928296 | 16645467 | 1.007085 | 0.640538677-1.583386369 | 0.9756049 | -0.8752107 | 2.46E-05 | -0.53501 | 0.000689 |
| *LINC01549* | ENSG00000232560 | 21 | 17438821 | 17450104 | 0.886177 | 0.782075399-1.004135338 | 0.0580608 | 2.99660181 | 3.26E-14 | 1.314478 | 8.49E-06 |
| *AP000472.1* | ENSG00000227075 | 21 | 21933315 | 21975681 | 1.119947 | 0.435208576-2.882022143 | 0.8142889 | -1.4494686 | 9.28E-05 | -1.26922 | 5.85E-06 |
| *AP001596.1* | ENSG00000232692 | 21 | 26378552 | 26471698 | 0.178368 | 0.001004099-31.68532648 | 0.5142032 | 1.90320731 | 2.93E-05 | 1.192065 | 5.61E-06 |
| *AP000695.2* | ENSG00000233818 | 21 | 36445731 | 36532408 | 0.919726 | 0.650371424-1.30063418 | 0.6360075 | 1.33283343 | 6.37E-07 | 0.768492 | 6.56E-06 |
| *DSCR9** | ENSG00000230366 | 21 | 37208503 | 37221736 | 152.0292 | 1.519381802-15212.03525 | 0.0325191 | 1.56608202 | 1.37E-08 | 0.590233 | 5.69E-05 |
| *AP001432.1* | ENSG00000242553 | 21 | 37221419 | 37237744 | 1.802212 | 0.764016069-4.25117709 | 0.1785523 | 1.82183379 | 7.43E-10 | 0.714416 | 9.28E-06 |
| *AP001626.1* | ENSG00000235023 | 21 | 42648271 | 42651244 | 1.207015 | 0.235652597-6.182343362 | 0.8213984 | 1.86005485 | 7.47E-05 | 1.117977 | 0.000464 |
| *AP001628.1* | ENSG00000225218 | 21 | 42831040 | 42836477 | 2.028701 | 0.806088627-5.105675753 | 0.1330445 | 1.6502426 | 6.92E-07 | 0.643816 | 0.000124 |
| *AP001065.3* | ENSG00000274225 | 21 | 44477850 | 44478493 | 1.031716 | 0.931475647-1.142744104 | 0.5493421 | -1.0523478 | 2.52E-05 | -1.27501 | 2.25E-13 |
| *LINC00205** | ENSG00000223768 | 21 | 45288050 | 45297806 | 1.175219 | 1.021455635-1.35212849 | 0.0240279 | 1.55020362 | 9.89E-24 | 0.744848 | 4.90E-13 |
| *LINC00316* | ENSG00000237664 | 21 | 45338590 | 45341990 | 0.959949 | 0.314121712-2.933583359 | 0.9428281 | 2.38526878 | 1.23E-05 | 1.309341 | 0.000112 |
| *LINC01694* | ENSG00000233922 | 21 | 45593654 | 45603088 | 1.428981 | 0.968168943-2.109122294 | 0.0723188 | 2.76519561 | 4.68E-11 | 1.525758 | 3.51E-07 |
| *AP001469.3** | ENSG00000239415 | 21 | 46251549 | 46254133 | 1.826543 | 1.141534718-2.922608599 | 0.0120076 | 1.96105566 | 1.03E-24 | 0.536465 | 5.11E-05 |
| *DGCR9* | ENSG00000273032 | 22 | 18970525 | 19031242 | 0.905864 | 0.443671862-1.849539622 | 0.7860308 | 2.07760413 | 2.23E-12 | 0.783898 | 0.000163 |
| *AL022324.3* | ENSG00000272942 | 22 | 25434324 | 25435070 | 2.315439 | 0.499402703-10.73533643 | 0.2833692 | 2.55177943 | 2.18E-06 | 1.217849 | 0.000266 |

| | | | | | | | | | | | |
|---|---|---|---|---|---|---|---|---|---|---|---|
| *AL008721.2* | ENSG00000272977 | 22 | 25476218 | 25479971 | 1.791725 | 0.603493841-5.31948796 | 0.2935486 | 1.22303631 | 1.19E-09 | 0.663065 | 9.28E-07 |
| *AC004832.5* | ENSG00000273350 | 22 | 30420512 | 30420912 | 0.957782 | 0.772448025-1.187583952 | 0.6942311 | -0.9281883 | 3.73E-05 | -0.7138 | 1.66E-05 |
| *AC004832.6* | ENSG00000273428 | 22 | 30435544 | 30436247 | 1.935338 | 0.248561718-15.06882548 | 0.5283278 | -0.8616625 | 0.000154 | -0.71186 | 3.60E-05 |
| *AC005005.3* | ENSG00000273387 | 22 | 31082156 | 31083565 | 1.316101 | 0.764207563-2.266557957 | 0.3219975 | 1.09925902 | 6.61E-08 | 0.533975 | 0.000122 |
| *Z97056.1* | ENSG00000228620 | 22 | 38424288 | 38427336 | 1.018431 | 0.941233204-1.101961194 | 0.6497551 | 1.53811614 | 4.27E-06 | -0.99677 | 5.45E-05 |
| *LINC01315* | ENSG00000229891 | 22 | 42364390 | 42369284 | 1.016622 | 0.779247368-1.326306645 | 0.9032861 | 1.37787392 | 1.69E-10 | 0.58864 | 0.000132 |
| *FIRRE** | ENSG00000213468 | X | 131688779 | 131830862 | 3.215623 | 1.023169703-10.10607467 | 0.0455901 | 3.27265568 | 3.51E-19 | 0.798581 | 0.000355 |
| *LINC00632* | ENSG00000203930 | X | 140709562 | 140793215 | 1.833973 | 1.198149366-2.80720913 | 0.0052339 | 2.66432384 | 2.91E-05 | 2.004211 | 1.02E-06 |
| *AC244090.3* | ENSG00000261773 | X | 154517840 | 154518631 | 6.424419 | 0.660925747-62.44750699 | 0.1089185 | 1.60849429 | 7.26E-05 | 0.826078 | 0.000293 |
